# QMEANDisCo—distance constraints applied on model quality estimation

**DOI:** 10.1093/bioinformatics/btz828

**Published:** 2019-11-07

**Authors:** Gabriel Studer, Christine Rempfer, Andrew M Waterhouse, Rafal Gumienny, Juergen Haas, Torsten Schwede

**Affiliations:** 1 Biozentrum, University of Basel, Basel 4056, Switzerland; 2 SIB Swiss Institute of Bioinformatics, Basel 4056, Switzerland

## Abstract

**Motivation:**

Methods that estimate the quality of a 3D protein structure model in absence of an experimental reference structure are crucial to determine a model’s utility and potential applications. Single model methods assess individual models whereas consensus methods require an ensemble of models as input. In this work, we extend the single model composite score QMEAN that employs statistical potentials of mean force and agreement terms by introducing a consensus-based distance constraint (DisCo) score.

**Results:**

DisCo exploits distance distributions from experimentally determined protein structures that are homologous to the model being assessed. Feed-forward neural networks are trained to adaptively weigh contributions by the multi-template DisCo score and classical single model QMEAN parameters. The result is the composite score QMEANDisCo, which combines the accuracy of consensus methods with the broad applicability of single model approaches. We also demonstrate that, despite being the *de-facto* standard for structure prediction benchmarking, CASP models are not the ideal data source to train predictive methods for model quality estimation. For performance assessment, QMEANDisCo is continuously benchmarked within the CAMEO project and participated in CASP13. For both, it ranks among the top performers and excels with low response times.

**Availability and implementation:**

QMEANDisCo is available as web-server at https://swissmodel.expasy.org/qmean. The source code can be downloaded from https://git.scicore.unibas.ch/schwede/QMEAN.

**Supplementary information:**

Supplementary data are available at *Bioinformatics* online.

## 1 Introduction

Modelling methods, in particular homology/comparative modelling, have established themselves as a valuable complement to structural analysis when experimental data are missing (Schmidt *et al.*, 2014). While such methods have matured into pipelines that can generate models for almost any protein automatically, the quality of the generated models can be highly variable and hard to predict in the absence of experimental observables. This is a major concern as the range of applications for which the model can be used directly depends on its quality (Baker and Sali, 2001; Schwede, 2013), hence the importance of quality estimation methods. Quality estimates can be global full-model estimates, e.g. to pick the best model in a set of alternatives, or local per-residue estimates. The latter allows for a more specific model selection in cases where only one particular part of the protein is of interest, e.g. a domain containing an active site. It can also guide the modelling process itself, for instance detecting regions requiring further refinement or choosing from alternative local conformations.

Current approaches to the quality estimation problem can be classified in single model methods, consensus methods and quasi-single model methods. Most single model methods use knowledge-based approaches to express the expected similarity to the actual native structure with a numeric value (Benkert *et al.*, 2011; Olechnovič and Venclovas, 2017; Uziela *et al.*, 2017). Such methods have the advantage of only requiring a single model as input, but they are often outperformed by consensus methods that base their prediction on an ensemble of models (Kryshtafovych *et al.*, 2016, 2018). Model quality is estimated from the variability of the models in the ensemble, assuming that correct structural features will tend to be more conserved (Cao *et al.*, 2014; Ginalski *et al.*, 2003; Skwark and Elofsson, 2013). Nevertheless, their application is somewhat limited as a well-defined independent set of models may not always be available in many applied cases. An alternative is so called quasi-single model methods (Maghrabi and McGuffin, 2017), which try to combine the convenience of taking a single model as input with the predictive power of consensus methods by generating ensemble information themselves. From a user perspective, quasi-single model methods can be considered single model methods, as they are independent from external ensemble information as input.

**Fig. 1.**
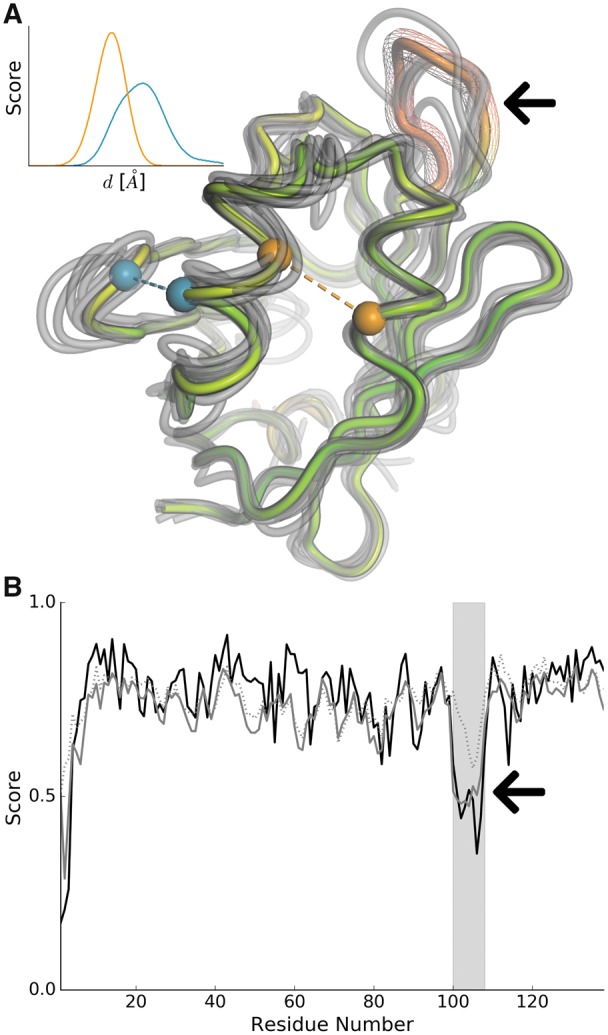
Example application of distance constraints on model quality estimation. (**A**) Per-residue QMEANDisCo scores are mapped as red-to-green colour gradient on a model of lbp-8 (lipid binding protein) in *Caenorhabditis elegans* (UniProtKB: O02324, PDB: 6C1Z). Distance constraints have been constructed from an ensemble of experimentally determined protein structures that are homologous to lbp-8. A representative subset of homologues is illustrated in grey. The inset depicts two example constraints between residues marked with colour-coded spheres in the model. (**B**) The solid black line represents per-residue lDDT scores of the model given the known target structure. Grey dots represent QMEANDisCo if only single model scores are employed. Adding distance constraints (solid grey line) helps to identify deviations in the loop marked with an arrow in A and B. (Color version of this figure is available at *Bioinformatics* online.)

One established single model method is QMEAN (Benkert *et al.*, 2008, 2011). QMEAN is a composite score employing statistical potentials of mean force (Sippl, 1993) and the consistency of a model with structural features predicted from sequence. We introduce a new distance constraint (DisCo) score that assesses the agreement of pairwise distances in a model and an ensemble of constraints derived from experimentally determined protein structures that are homologous to the model being assessed. DisCo can considered to be a quasi-single model score as it derives its own ensemble information with a sequence based homology search as illustrated in Figure 1. Using homologous structures directly, allows keeping the computation time low as no models must be built. This work combines DisCo with the single model scores from QMEAN. In case many close homologues exist, DisCo is expected to be very accurate. However, accuracy declines if few or no close homologues can be identified. In order to combine the ability of single model scores to assess individual models with the power of DisCo in cases of sufficient structural information, we use feed-forward neural networks to adaptively weigh the various components. The result is a composite score for accurate local and global model quality estimates: QMEANDisCo.

A previous version of QMEANDisCo (version 2) is briefly described in Waterhouse *et al.* (2018). This manuscript describes QMEANDisCo in detail, including recent improvements leading to the current version 3. QMEANDisCo is continuously evaluated in the CAMEO continuous evaluation platform (Haas *et al.*, 2018), where it currently ranks best among the registered methods while having the lowest response time. Furthermore, QMEANDisCo participated in the 13th round of the CASP experiment (Moult *et al.*, 2018) under the working title FaeNNz and ranked among the top performers.

## 2 Materials and methods

### 2.1 Target value

We use the lDDT score (Mariani *et al.*, 2013) in range [0.0, 1.0] as target value for local and global quality estimates. lDDT is a superposition free score and assesses differences in pairwise interatomic distances between model and reference structure on a full-atomic basis. Only distances up to 15 Å are considered, reducing the effect of domain movement events. lDDT very closely agrees with other ‘local scores’, such as CAD (Olechnovič *et al.*, 2013) or RPF (Huang *et al.*, 2012; Olechnovič *et al.*, 2019). Prediction performance can thus expected to be comparable for this full group of scores. We deliberately avoid scores based on reduced structural representations since they do not reflect the wide variety of local interactions in sufficient detail (Haas *et al.*, 2018). These types of scores include Cα distance based per-residue measures that are obtained after a global superposition of model and target.

### 2.2 Evaluation methods

The focus of this work lies on predicting per-residue quality estimates. For evaluation, we rely on receiver operating characteristic (ROC) analysis, which is common in the field (Haas *et al.*, 2018; Kryshtafovych *et al.*, 2016, 2018). ROC allows visualizing a predictors’ ability of distinguishing correctly and incorrectly modelled residues and quantifying the outcome with the area under the curve (AUC). Single residue data points with lDDT >0.6 are classified as correctly modelled.

ROC analysis is performed by pooling all per-residue data points from a full test set to get an overall AUC, which mainly analyses the ability of assigning absolute quality estimates. Additionally, it is performed on a per-model basis. To quantify the outcome of the latter, the mean per-model AUC, the expected AUC when looking at one particular model, is used as performance indicator. Models where all residues are classified equally (e.g. all classified as ‘incorrect’) lead to invalid ROC AUC values and are excluded from the mean calculation.

### 2.3 Single model scores

The single model scores from the current version of QMEAN (3) form the basis to obtain per-residue scores in QMEANDisCo. They are suitable for assessing individual models and are summarized here with their respective statistical potential of mean force terms parametrized as further described in the Supplementary Materials:

- All-atom interaction potential: pairwise interactions are assessed between all chemically distinguishable heavy atoms. A sequence separation threshold has been introduced to reduce contributions from residues adjacent in sequence thereby focussing on long-range interactions and reduce the effect of local secondary structure.
- Cβ interaction potential: this term assesses the overall fold by only considering pairwise interactions between Cβ positions of the 20 default proteinogenic amino acids. In case of glycine, a representative of Cβ is inferred from the N, Cα and C backbone atom positions. The same sequence separation as in the all-atom interaction potential is applied.
- Packing potential: assesses the number of surrounding atoms around all chemically distinguishable heavy atoms not belonging to the assessed residue itself.
- Torsion potential: the central Φ/Ψ angles of three consecutive amino acids are assessed based on the identities of the triplet using a grouping scheme described by Solis and Rackovsky (2006).
- Solvent accessibility agreement: binary classification whether solvent accessibility of a residue matches with the prediction from ACCpro (Cheng *et al.*, 2005).
- Secondary structure agreement: a log-odds score that relates the probability of observing a DSSP state (Kabsch and Sander, 1983) in combination with a PSIPRED prediction (Jones, 1999) with the probability of observing the two events independently of each other (Soding, 2005)**:** *S(d, p, c)=log[p(d, p, c)/(p(d)p(p, c))]* with *d* representing a DSSP state in [G, H, I, E, B, T, S, C], *p* a PSIPRED state in [H, E, C] and *c* a PSIPRED confidence value in [0–9].
- Solvent accessibility: accessibility in Å^2^ as calculated with DSSP, scaled to [0, 1].

QMEANDisCo (3) introduces four additional terms. The first two of them are statistical potentials of mean force that are parametrized as described in the Supplementary Materials.

- Cβ packing potential: same concept as the packing potential, but only Cβ atoms are considered. Glycine is treated the same way as in the Cβ interaction potential.
- Reduced potential: assesses pairwise interactions between reduced representations of amino acids. The reduced representation is composed of the Cα position and a directional component constructed from backbone N, Cα and C positions with further details available in the Supplementary Materials. As in the two other interaction potentials, a sequence separation threshold is applied.
- Clash score: full-atomic clash score as defined for SCWRL3 (Canutescu *et al.*, 2003).
- *N*: number of residues within 15 Å by using Cα atoms as reference positions.

The scores are evaluated on a per-residue basis with full-atomic scores averaging their per-atom contributions. Before further processing, all per-residue scores except the number of residues (*N*) undergo a spherical smoothing (*σ* = 5 Å) as described for QMEANBrane (Studer *et al.*, 2014).

### 2.4 DisCo

DisCo is derived from QMEANDist, a quasi-single model method that participated in the CASP9 experiment as a global quality predictor (Biasini, 2013; Kryshtafovych *et al.*, 2011). We revisited the approach of assessing the agreement of pairwise residue–residue distances with ensembles of constraints extracted from experimentally determined protein structures that are homologous to the assessed model. Instead of generating global quality estimates, DisCo aims to predict local per-residue quality estimates. After extracting the target sequence of the model to be assessed, homologues are identified using HHblits (Remmert *et al.*, 2011, the used command line arguments are available in the Supplementary Materials). For each homologue *k*, all Cα positions are mapped onto the target sequence using the HHblits alignment. Gaussian distance constraints for residue pairs (*i, j*) are generated for all Cα–Cα distances *μ_ijk_* below 15 Å:

(1) $$g_{\mathrm{ijk}}(d_{\mathrm{ij}})\;=\;\;\text{exp}[-\frac{1}{2}(d_{\mathrm{ij}}-\;\mu_{\mathrm{ijk}}){}^{2}]$$

The goal is to construct a pairwise scoring function *s_ij_*(*d_i__j_*), that assesses the consistency of a particular pairwise Cα–Cα distance *d_ij_* in the model with all corresponding constraints *g_ijk_*(*d_ij_*). In order to avoid biases towards overrepresented sequence families among all found homologues, they are clustered based on their pairwise sequence similarity as specified in the Supplementary Materials. Since the templates often do not cover the entire target sequence, some Cα–Cα pairs might not be represented in every template and consequently the number of templates *n_ijc_* containing a Cα–Cα pair varies within a cluster for different (*i, j*). Only if a Cα–Cα pair is present in a cluster c, we construct a cluster scoring function *h_ijc_*(*d_ij_*):

(2) $$h_{\mathrm{ijc}}(d_{\mathrm{ij}})=\frac{1}{n_{\mathrm{ijc}}}\sum_{k\in c}g_{\mathrm{ijk}}(d_{\mathrm{ij}})$$

**Fig. 2.**
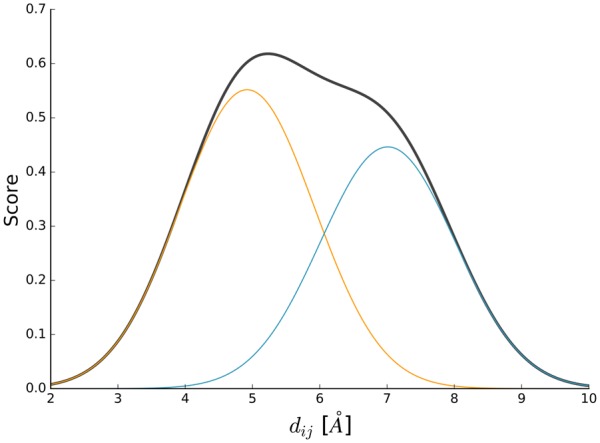
Example DisCo scoring function. The pairwise scoring function *s_ij_*(*d_ij_*) (black) represents the Cα–Cα distance between residues *i* and *j*. It is composed of two weighted cluster scoring functions *w_c_h_ijc_*(*d_ij_*) with weights determined from avg. cluster sequence similarities (*SS_c_*) 0.276 (orange) and 0.273 (blue). (Color version of this figure is available at *Bioinformatics* online.)

To get the desired pairwise scoring function *s_ij_*(*d_ij_*) we combine *h_ijc_*(*d_ij_*) from each cluster *c* in a weighted manner as exemplified in Figure 2. Clusters expected to be closely related to the target sequence contribute more than others:

(3) $$s_{\mathrm{ij}}(d_{\mathrm{ij}})=\sum_{c}w_{c}h_{\mathrm{ijc}}(d_{\mathrm{ij}})$$

with weights *w_c_* defined as *exp*[*γSS_c_*] and normalized, so that the weights of all clusters in which the Cα–Cα pair is present, sum up to one. *SS_c_* is the average normalized sequence similarity towards the target sequence of cluster *c* and *γ* is a constant that controls how fast the influence of a cluster vanishes as a function of *SS_c_*. The default value for *γ* is 70 and the effect of varying *γ* is discussed in Supplementary Figure S3. The DisCo score of a single residue of the model at position *i* then is computed by averaging the outcome of all *n* pairwise scoring functions *s_ij_*(*d_ij_*) towards other residues *j ≠ i* with their Cα positions within 15 Å:

(4) $${\mathrm{DisCo}}_{i}=\frac{1}{n}\sum_{j}s_{\mathrm{ij}}\left(d_{\mathrm{ij}}\right).$$

As the accuracy of DisCo depends on the underlying templates, features describing its reliability are required to optimally weigh DisCo with the single model scores in a subsequent machine-learning step. For each residue *i* there are:

- Average number of clusters *c* of each evaluated pairwise function *s_ij_*(*d_ij_*).
- Average sequence similarity *SS* of each evaluated pairwise function *s_ij_*(*d_ij_*) with *SS* being defined as the maximum *SS_c_* of all underlying clusters *c*.
- Same as above but for sequence identities.
- Average variance *v* of each evaluated pairwise function *s_ij_*(*d_ij_*) with *v* being the variance of all observed distances in any of the underlying clusters *c*.
- Number of evaluated pairwise functions *s_ij_*(*d_ij_*).
- Total number of pairwise functions for residue *i*.
- The fraction between the previous two items.

### 2.5 Score combination

Fully connected feed-forward neural networks are used to learn complex interdependencies of scores described in the two previous sections. Furthermore, they adaptively weigh single model scores that are capable of scoring individual models and DisCo that depends on dynamically generated constraint data. Neural network training and validation relied on two datasets:

- CAMEO: all models submitted to the CAMEO QE category during 1 year (CAMEO weeks from July 1, 2017 to June 30, 2018) have been collected. This results in a set of ∼2.4 million per-residue data points from 9500 models built for 883 unique targets. DisCo scores for this set have been estimated from SWISS-MODEL HHblits template searches performed at the time of the CAMEO submission and thus do not contain the target structure.
- CASP12: the CASP12 EMA category (Kryshtafovych *et al.*, 2018) submitted models for each target in two stages. ‘Stage 1’ was a selection of 20 models and ‘stage 2’ the 150 best models according to the Davis–EMA consensus baseline predictor. For each of the 70 finally evaluated targets, 101 of the 150 models submitted in ‘stage 2’ have randomly been selected. For the CASP12 dataset, this results in ∼1.9 million per-residue data points from 7070 models built for 70 unique targets. DisCo scores for this set have been estimated from HHblits template searches where every template with a release date after May 1, 2016 has been discarded.

Neural networks predicting per-residue lDDT scores are trained with the tiny-dnn deep learning framework (https://github.com/tiny-dnn/tiny-dnn). The objective is to identify optimal training parameter/network topology combinations among the following: error functions: [‘absolute’, ‘mse’]; optimizing algorithms: [‘adam’, ‘rmsprop’]; training epochs: [100, 200, 400]; training batch sizes: [100, 200, 400]; network topologies: [[*n*, 20, 20, 20, 1], [*n*, 40, 20, 20, 1], [*n*, 40, 20, 10, 1], [*n*, 40, 40, 40, 1], [*n*, 80, 80, 80, 1], [*n*, 80, 40, 20, 1], [*n*, 80, 40, 20, 20, 1], [*n*, 40, 40, 20, 20, 1], [*n*, 40, 20, 20, 10, 1]]. ‘ReLu’ has been used as activation function.

The size of the input layer is denoted with *n*. There are 11 per-residue features as described in Section 2.3 and 8 as described in Section 2.4. Eight features are full-model averages of the statistical potential and agreement terms in Section 2.3. Amino acid specific biases for composite scores have been identified in previous work (Studer *et al.*, 2014) and taken into account in the input layer by using ‘one-hot’ encoding. This adds another 20 features, one for each standard proteinogenic amino acid. The input layer thus comprises *n *=* *47 nodes if all input features are valid. All data points with one or more invalid input features have been excluded in a first stage. Examples are terminal residues for which no backbone dihedral angles can be estimated or invalid DisCo scores due to lacking HHblits search results.

A 5-fold cross-validation (80% training, 20% validation) has been constructed with the data split on the level of modelling targets. A cross-validation means to evaluate five validation sets with five neural networks that did not use any data from the corresponding validation set for training. The predictions for all five validation sets are pooled together and evaluated at once. We allow not only performing a classical cross-validation on one dataset (e.g. CAMEO) but also across datasets (e.g. training on CAMEO and validation on CASP12).

A general trend for more difficult modelling targets in CASP (Haas *et al.*, 2018), as well as more diverse modelling approaches, lead to significantly different per-residue lDDT distributions in the CAMEO and CASP12 datasets (Supplementary Fig. S1). This may introduce biases in the training procedure. A mixed dataset only for training has therefore been constructed. For all five pairs of CAMEO and CASP12 training sets, a mixed training set has been constructed by randomly selecting 50% of the data points from the other two. This results in a valid cross-validation across datasets (e.g. training on mixed and validation on CASP12).

Networks for all training parameter/network topology combinations have been trained on all cross-validation training sets (CAMEO, CASP12, mixed) and validated on CAMEO and CASP12. Both validations lead to a separate ranking of networks by the mean per-model AUC as described in Section 2.2. The intersection of the top 100 from both rankings, a set of 59 training parameter/network topology combinations (Supplementary Table S1), is considered further.

The problem of missing input data was approached by implementing a neural network scorer (NNScorer). The NNScorer is comprised of a full ensemble of networks with equal training parametrization and network topology, except the number of input nodes *n*. The intention is to provide a network for each potential combination of valid input features, so called feature groups that are defined in the Supplementary Materials. There are 16 feature groups and thus 16 networks per NNScorer. NNScorers have been trained for the 59 successful training set/training parameter/network topology combinations selected in the first validation round.

A second optimization round has been performed considering all data points of the validation sets, as invalid input features can now be handled by the NNScorer. Again, the validations on CAMEO and CASP12 have separately been ranked by the expected per-model AUC (Supplementary Table S2). The following has been found to be a good compromise when validated on both, CAMEO and CASP12: training data: mixed; error function: ‘mse’; optimizing algorithm: ‘rmsprop’; training epochs: 100; training batch size: 400; network topology: [*n*, 20, 20, 20, 1]. The finally used NNScorer has been re-trained with this parametrization/topology using the full mixed set for training.

To conclude, the cross-validation performance was increased when replacing the linear combination of QMEAN 3 with neural networks and incorporating features specific to QMEANDisCo (see Supplementary Tables S3 and S4).

Furthermore, no training parameter/network topology combination has reached the second round of validation when CASP12 has been used for training. To thoroughly investigate the effect of training data, the two rounds of parameter/topology optimization described in this section have been repeated only with CASP12 as training data. The results in Supplementary Table S5 confirm that the finally used NNScorer trained on mixed performs better than any NNScorer trained on CASP12 even when validated on CASP12.

### 2.6 Full-model scoring

Given the nature of lDDT, the average of accurate per-residue quality estimates can be expected to be a good approximation of the global overall quality. That is the definition of the QMEANDisCo global score. The expected error of the global score prediction is defined as the root mean square deviation of prediction and actual global lDDT on a large set of models. As this is derived from the global scoring evaluation, it will further be discussed in Section 3.5.

### 2.7 Blind test data

All data used for testing/benchmarking were obtained through regular blind predictions from QMEAN-Server instances registered to CAMEO and CASP13. For CASP13, we registered a private QMEAN-Server instance. The server initially deployed a development method called FaeNNz. FaeNNz is conceptually equivalent to QMEANDisCo 3 with the key difference of using less data from CAMEO in the training procedure described in Section 2.5 (data collected during 10 months instead of 1 year). Furthermore, FaeNNz adequately reflects the performance of the final version of QMEANDisCo as demonstrated with a retrospective analysis of QMEANDisCo 3 on CASP13 data (Supplementary Table S7). Test data collection in detail:

- CAMEO: per-residue predictions are evaluated on data collected from ∼3000 models during a time range of 12 weeks for 13 registered methods (CAMEO submission dates December 1, 2018–March 3, 2019). To have enough data to estimate the expected error described in Section 2.6, data from additional ∼4500 models had been collected during 20 weeks for global scoring analysis (CAMEO submission dates July 7, 2018–November 24, 2018).
- CASP13: the CASP13 EMA category submitted models for each target in two stages. ‘Stage 1’ was a selection of 20 models and ‘stage 2’ the 150 best models according to the Davis–EMA consensus baseline predictor. Per-residue predictions are evaluated on data from the full set of ∼12000 models submitted as ‘stage 2’ for 80 unique targets for all registered methods that submitted at least 50% of the requested per-residue predictions (27 methods). To evaluate global full-model predictions we refer to the publicly available automated analysis provided by the CASP13 assessors.

### 2.8 Implementation and availability

The QMEAN-Server (https://swissmodel.expasy.org/qmean) makes QMEANDisCo accessible to non-expert users with the option to access it through an application programming interface. Alternatively, the underlying source code can be downloaded from https://git.scicore.unibas.ch/schwede/QMEAN under the permissive Apache v2.0 license. The software is based on the OpenStructure computational structural biology framework (Biasini *et al.*, 2010, 2013). Computationally intensive tasks are implemented in C++ and exported to the Python scripting language to increase flexibility and speedup prototyping of new quality estimation algorithms.

## 3 Results

### 3.1 Overall AUC analysis

**Fig. 3.**
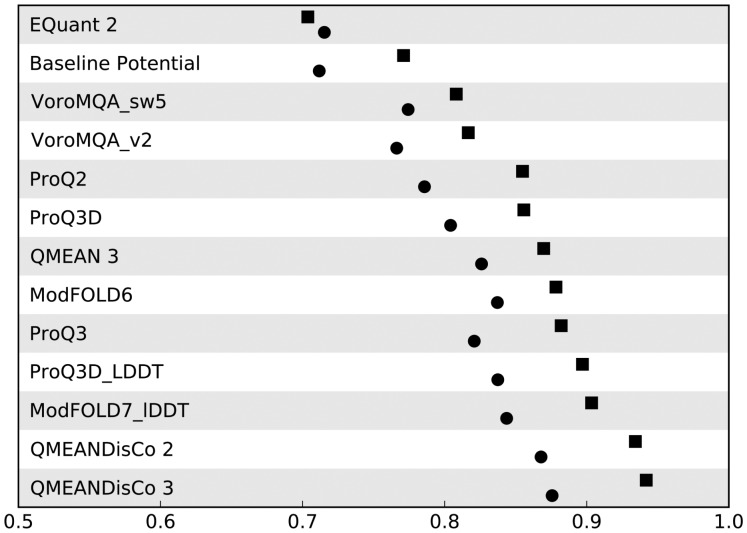
Evaluation of per-residue quality estimates on CAMEO data. Squares represent performance in terms of overall AUC and circles the mean per-model AUC. The raw data are available in Supplementary Table S6. ‘QMEANDisCo 2’ denotes an initial version of QMEANDisCo briefly described in Waterhouse *et al.* (2018), ‘QMEANDisCo 3’ denotes the method described in this manuscript

**Fig. 4.**
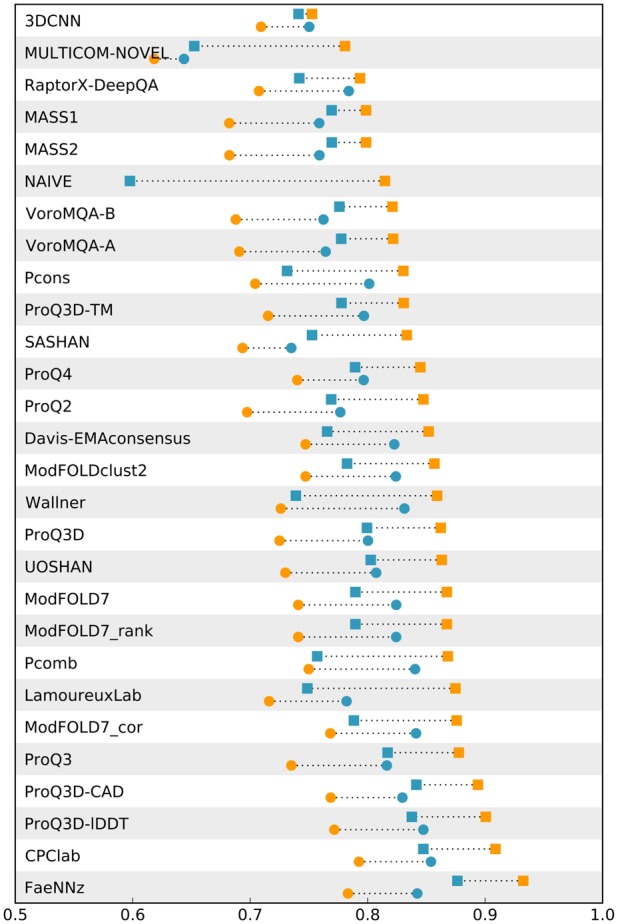
Evaluation of per-residue quality estimates on CASP13 data. Squares represent performance in terms of overall AUC and circles the mean per-model AUC. Orange colours stand for results on the full dataset and blue for results from a subset composed of high quality models with global lDDT >0.6. FaeNNz represents QMEANDisCo 3 and NAIVE is the naive predictor described in Section 3.2. Raw data are available in Supplementary Table S7. (Color version of this figure is available at *Bioinformatics* online.)

Comparing QMEAN and QMEANDisCo on CAMEO per-residue data reveals large improvements in overall AUC (0.87 versus 0.94) when adding the described scores and enhanced machine-learning techniques (Fig. 3, Supplementary Table S6). This is consistent with the observed improvements during training (Supplementary Table S3). On both test sets, CAMEO and CASP13, QMEANDisCo, FaeNNz, respectively, have the highest overall AUC among all methods (Figs 3 and 4, Supplementary Tables S6 and S7).

### 3.2 Global effect on overall AUC

Already during training, the substantially different target value distribution of the used training sets was a concern. Similar distributions can be observed for the test sets (Supplementary Fig. S2). CASP13 has many low quality data points largely originating from random coil models. This gives rise to the hypothesis that much of the overall AUC performance could already be retrieved by detecting those random coils and predicts all their residues to be of low quality. To test this hypothesis, a naive predictor for CASP13 has been implemented. The global full-model score of the Davis–EMA consensus baseline predictor is blindly assigned to each residue of a model. Detecting random coils and scoring their residues accordingly is not necessarily a bad idea, but this implementation has the obvious flaw of not being able to discriminate correctly and wrongly modelled residues in one particular model. The naive predictor performs surprisingly well with an overall AUC value of 0.82 (Fig. 4, Supplementary Table S7). This observation suggests that a good performance in terms of overall AUC might not solely be the result of assigning meaningful per-residue scores but to some extent also a global effect. Consequently, we extended our evaluation to include per-model performance indicators and, for CASP13, repeated it on a subset composed of high quality models.

### 3.3 Per-model analysis

As for the overall AUC, QMEANDisCo performs best for the per-model AUC on CAMEO (Fig. 3, Supplementary Table S6). On CASP13, CPClab (Mulnaes and Gohlke, 2018) slightly outperforms FaeNNz (per-model AUC of 0.79 versus 0.78) (Fig. 4, Supplementary Table S7). Also ModFOLD7_cor (Maghrabi and McGuffin, 2017), ProQ3D_LDDT and ProQ3D_CAD (Uziela *et al.*, 2018) exhibit no significant difference in per-model AUC (per-model AUC of 0.77 for all three) (Fig. 4, Supplementary Table S7).

### 3.4 Analysis on high quality models in CASP13

Overall and per-model AUC analyses on CASP13 have been repeated on a high quality subset composed of models with global lDDT >0.6. Per-residue data points from ∼20% of the original models remained and the target value distribution of the subset is similar to what can be observed for CAMEO (Supplementary Fig. S2). The performance of the naive predictor completely breaks down (Fig. 4). Simply detecting random coils does not work in this setup, as they are not prevalent anymore. A decline in overall AUC is observed for all methods with some affected more than others. FaeNNz still performs best with an overall AUC of 0.88 (Fig. 4, Supplementary Table S7).

The contrary can be observed for per-model AUC. All methods show increased performance which hints at general difficulties in discriminating well and poorly modelled residues in models of medium or low quality. FaeNNz ranks third, slightly outperformed by CPClab and ProQ3-LDDT. PComb (Wallner and Elofsson, 2007) and ModFOLD7_cor rank fourth and fifth with marginal lower per-model AUC (per-model AUCs of the top five methods: 0.85, 0.85, 0.84, 0.84 and 0.84) (Fig. 4, Supplementary Table S7).

### 3.5 Global scoring

**Fig. 5.**
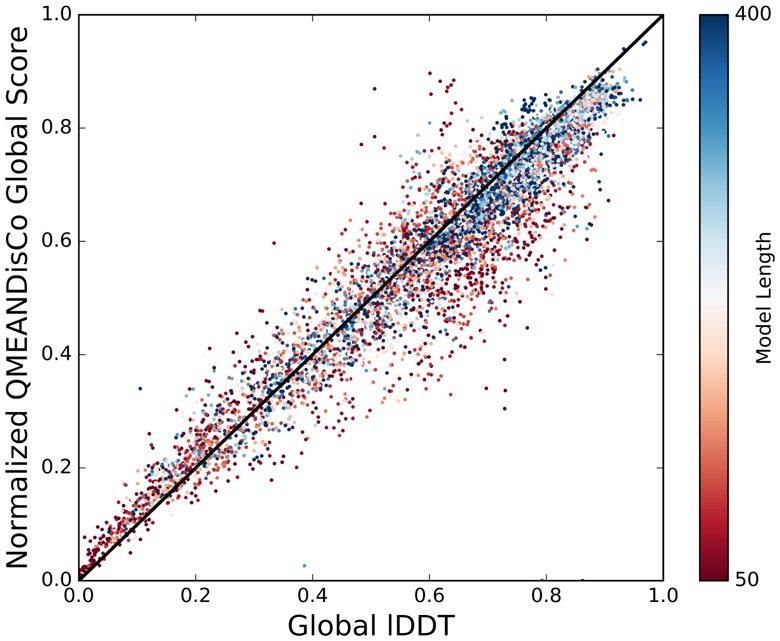
Evaluation of full-model quality estimates on CAMEO data. The QMEANDisCo global scores are length normalized to take the coverage dependency of the global lDDT score into account. Model length is depicted as colour gradient to emphasize increased prediction uncertainty when assessing small models. (Color version of this figure is available at *Bioinformatics* online.)

lDDT and other target values penalize for incomplete models. This is not the case for QMEANDisCo as it evaluates a model ‘as is’. The consequences are potential over-predictions in the CAMEO global scoring evaluation (Supplementary Fig. S4). The fraction of residues in the target structure that are covered in the model has therefore been applied as pre-factor of the QMEANDisCo global score for evaluation purposes. The result is an excellent correspondence between prediction and target reflected by a Pearson correlation coefficient of 0.95 (Fig. 5). The expected prediction error defined in Section 2.6 is based on these results and provided to the user of the QMEAN-Server. The expected error is not estimated on the full results but as a function of model length to emphasize increased uncertainties for smaller models (Fig. 5). For a model of length *l*, data points from models of length *l ± 40* are considered. The error for short models (40 residues) is expected to be ∼0.12 but quickly converges to ∼0.05 with increasing model size (Supplementary Table S8). This is consistent with the average absolute lDDT prediction error of FaeNNz in CASP13 (∼0.05 for ‘stage 1’ and ∼0.06 for ‘stage 2’, http://predictioncenter.org/casp13/qa_diff_mqas.cgi).

In CASP13, FaeNNz global scores were normalized based on model length and known target sequence. Table 1 displays a selection of top performing methods with respect to ROC AUC as calculated by the CASP13 assessors for global full-model scoring evaluation. In addition to lDDT, ROC AUC values are also displayed for GDT_TS (Zemla, 2003) and CAD (Olechnovič *et al.*, 2013). The results show a superior prediction performance of consensus methods for the superposition dependent GDT_TS score that only considers Cα positions. However, for the superposition independent full-atomic lDDT and CAD scores, the kind of scores we aim to predict, single model methods catch up. FaeNNz ranks second when considering lDDT and first when considering CAD.

**Table 1.** Global scoring ROC AUC analysis to evaluate the ability of discriminating good and bad models in CASP13

Method	GDT_TS	lDDT	CAD
Bhattacharya-ClustQ	0.942	0.949	0.932
**FaeNNz**	0.889	0.947	0.937
CPClab	0.909	0.936	0.928
MULTICOM_CONSTRUCT	0.945	0.931	0.914
MUfoldQA_T	0.961	0.929	0.909
**ProQ3D-CAD**	0.880	0.926	0.930
MUFoldQA_M	0.959	0.922	0.900
MULTICOM_CLUSTER	0.937	0.922	0.904
UOSHAN	0.959	0.917	0.898
**ProQ4**	0.875	0.913	0.907

*Note*: The data are extracted from http://predictioncenter.org/casp13/qa_aucmcc.cgi. The top 10 methods were selected according to a sorting by lDDT ROC AUC. ROC AUC values are displayed for lDDT and other scores relevant for CASP13 (GDT_TS, CAD). Single model methods (i.e. methods that need no ensemble of models as input) are highlighted with bold font, with FaeNNz (representing QMEANDisCo 3) conceptually being a quasi-single model method.

Another aspect discussed in the automated CASP13 results is model selection, i.e. given all models submitted for a target, what is the accuracy difference between the model selected by a quality estimation method and the optimal choice (http://predictioncenter.org/casp13/qa_diff2best.cgi). For QMEANDisCo (represented by FaeNNz), the lDDT difference for ‘stage 2’ is on average ∼0.07 as compared to ∼0.04 of the top performing method MULTICOM_CLUSTER (Hou *et al.*, 2019). From all 48 evaluated methods returning global scores, FaeNNz ranks 20th. For CAD, the average difference is ∼0.04 as compared to ∼0.03 from the top performer MULTICOM_CLUSTER which leads to rank eight.

## 4 Conclusion

In this work, we describe the QMEANDisCo composite score for single model quality estimation. It employs single model scores suitable for assessing individual models, extended with a consensus component by additionally leveraging information from experimentally determined protein structures that are homologous to the model being assessed. By using the found homologues directly, QMEANDisCo avoids the requirement of an ensemble of models as input.

To find the optimal combination of scores, we did profit from recent developments in the machine-learning community providing computational tools that efficiently learn complex interdependencies in large amounts of training data. However, careful data preparation and handling is crucial for optimal prediction performance. We showed that despite being the *de-facto* standard of benchmarking, CASP is not necessarily the ideal source of data to train predictive models. The situation was improved by combining data from CASP and CAMEO to increase training data diversity. The result was a predictive model that generalized the training data and provides accurate per-residue and full-model quality estimates for models of various origin. However, discriminating well and poorly modelled residues in protein models of lower overall quality remains a challenge.

QMEANDisCo has been developed with its application in the SWISS-MODEL homology modelling server in mind. A template search is the first step of any homology modelling pipeline. As this is the computationally most expensive step in QMEANDisCo, its integration into SWISS-MODEL comes at minimal additional computational cost. The low response times are also reflected in CAMEO where QMEANDisCo returns results within a few minutes with most of the time being spent in the template search step.

We believe that we provide a valuable tool that can easily be accessed through the QMEAN-Server. We demonstrated state-of-the-art performance in predicting lDDT scores with a focus on per-residue predictions. Prediction accuracy can expected to further increase given the growing number of experimentally determined protein structures (Bienert *et al.*, 2017).

## Supplementary Material

btz828_Supplementary_Data
