# QMEANDisCo—distance constraints applied on model quality estimation

**DOI:** 10.1093/bioinformatics/btaa058

**Published:** 2020-02-12

**Authors:** Gabriel Studer, Christine Rempfer, Andrew M Waterhouse, Rafal Gumienny, Juergen Haas, Torsten Schwede

**Affiliations:** 1 Biozentrum, University of Basel, Basel 4056, Switzerland; 2 SIB Swiss Institute of Bioinformatics, Basel 4056, Switzerland

*Bioinformatics* (2019) doi: 10.1093/bioinformatics/btz828

Correction text: The first version of this article placed the index of summation after, rather than below, the sum symbol in Equations 2, 3, and 4. This has now been corrected online. The publisher apologises for the error.

