# Supplementary material for: QMEANDisCo—distance constraints applied on model quality estimation
Source: Bioinformatics. 2019 Nov 7;36(6):1765–71. doi: 10.1093/bioinformatics/btz828 (PMC7075525; doi:10.1093/bioinformatics/btz828)
Supplement: btz828_Supplementary_Data [file btz828_supplementary_data.pdf]

QMEANDisCo – Distance Constraints Applied on Model Quality Estimation

Supplemental Materials

Gabriel Studer[1,2], Christine Rempfer[1,2], Andrew M Waterhouse[1,2], Rafal Gumienny[1,2], Juergen Haas[1,2] and Torsten Schwede[1,2]

[1]Biozentrum, University of Basel, Basel, 4056, Switzerland,

[2]SIB Swiss Institute of Bioinformatics, Basel, 4056, Switzerland

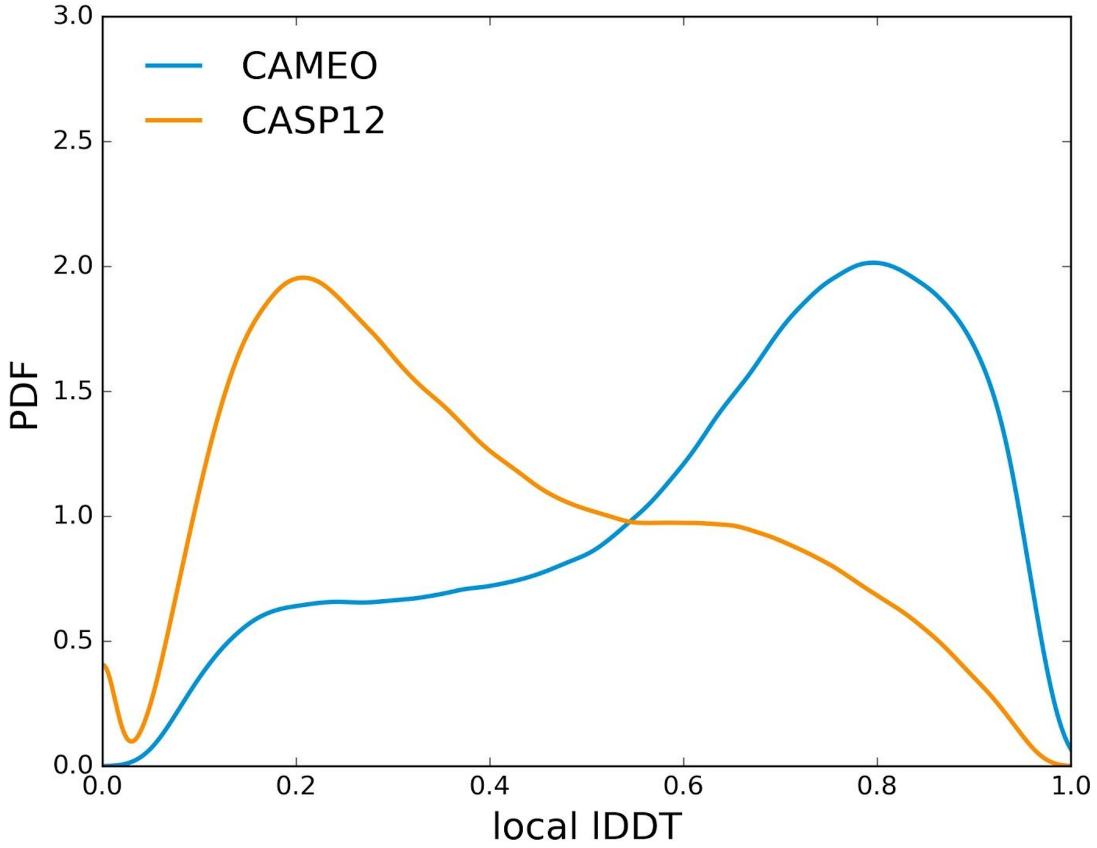

Fig. S1. Per-residue lDDT distribution of data sets used for training. Training relies on two data sets. One is compiled from data provided by the CASP12 experiment and another one from the CAMEO continuous evaluation platform. The probability density functions have been estimated with Gaussian kernel density estimates from raw per-residue lDDT data points. The peak at 0.0 for local lDDT arises from residues with stereo-chemical issues/clashes with the backbone atoms involved.

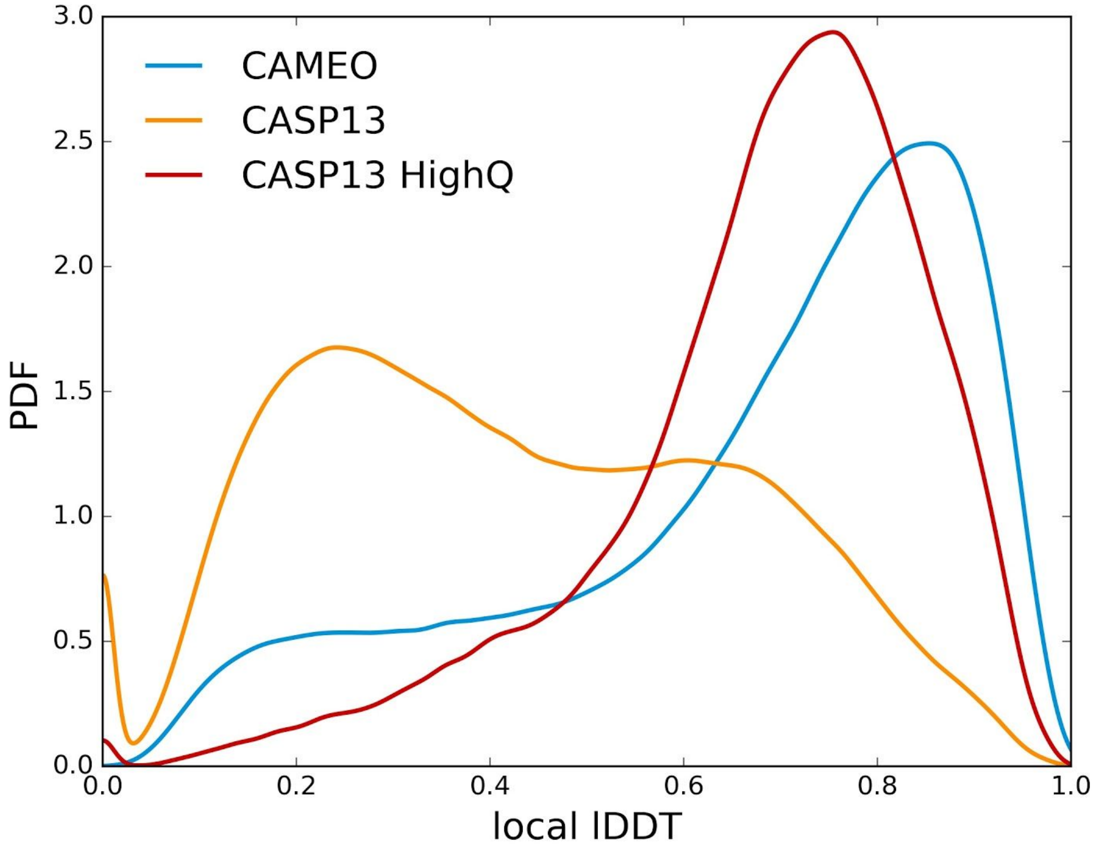

Fig. S2. Per-residue lDDT distribution of data sets used for testing. Testing relies on two data sets, one compiled from data provided by the CASP13 experiment and another one from the CAMEO continuous evaluation platform. An additional third set is a subset from CASP13 only originating from models with global lDDT > 0.6 (CASP13 HighQ). The probability density functions have been estimated with Gaussian kernel density estimates from raw per-residue lDDT data points. The peaks at 0.0 for local lDDT arise from residues with stereo-chemical issues/clashes with the backbone atoms involved.

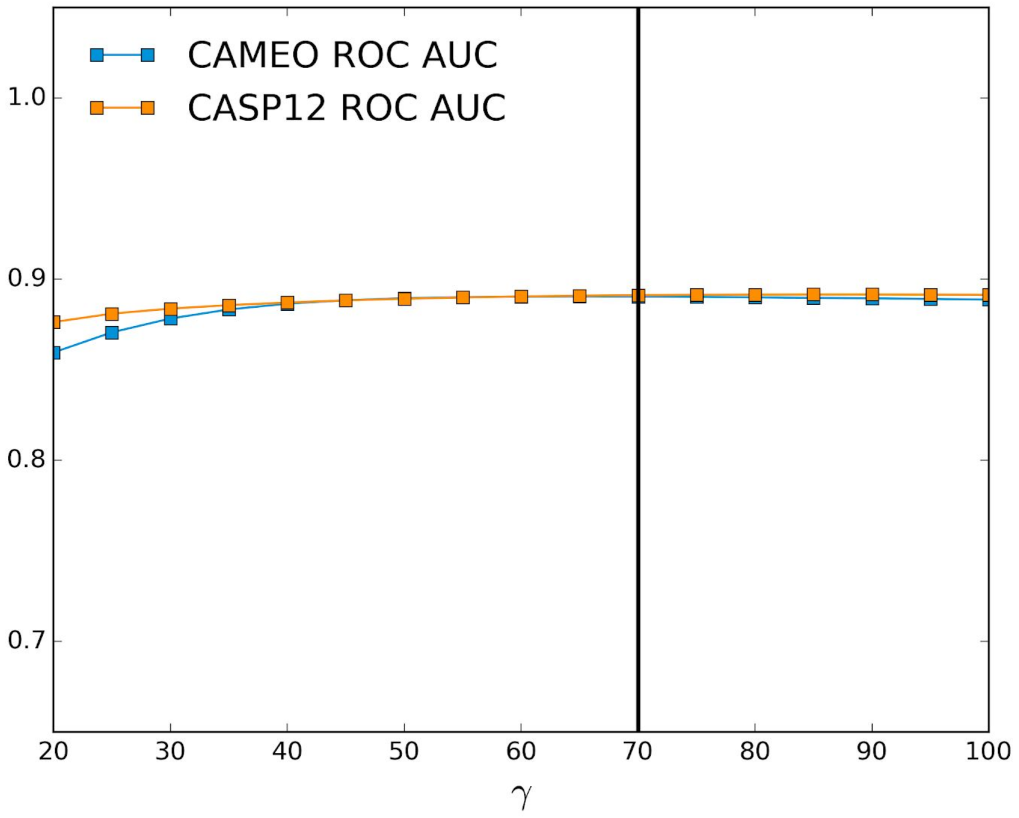

Fig. S3. Influence of γ on DisCo performance γ is relevant for constructing DisCo as described in section 2.4 of the main manuscript. Performance of DisCo with varying γ is estimated as ROC AUC with respect to per-residue lDDT scores. CAMEO and CASP12 are the data sets described in section 2.5 of the main manuscript and only per-residue data with valid DisCo score has been used for evaluation. The vertical line depicts the default value for γ (70).

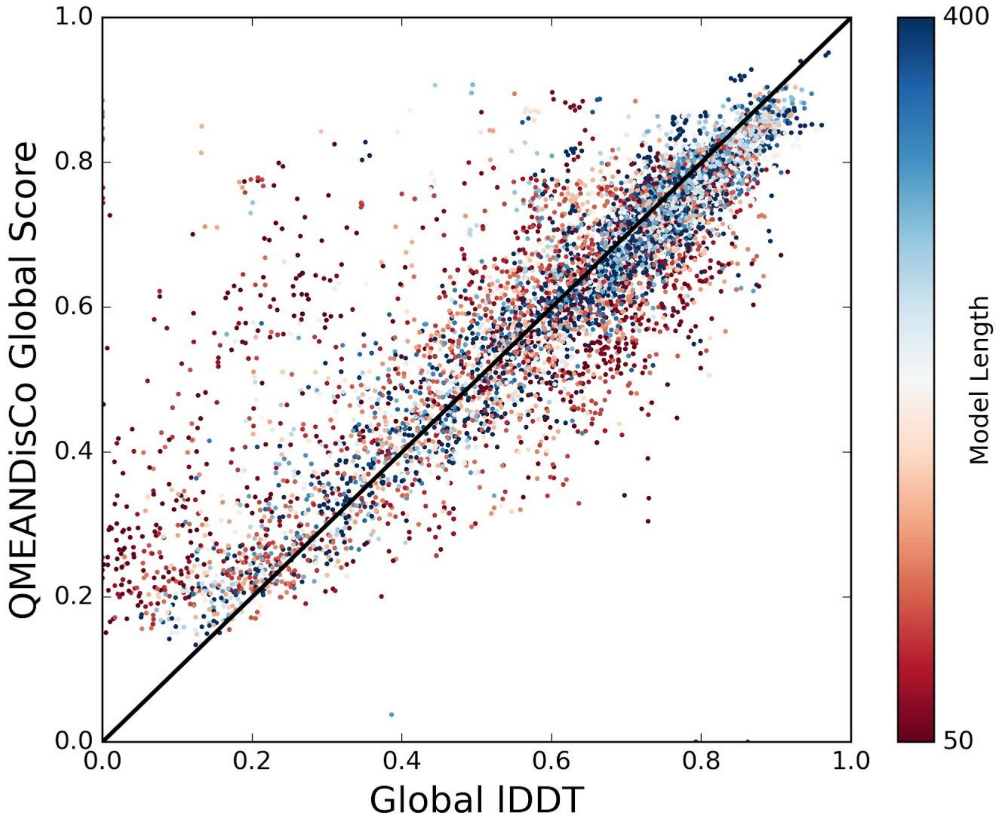

Fig. S4. Global scoring evaluation on CAMEO models. lDDT penalizes for missing residues. QMEANDisCo does not consider that by default and thus overpredicts incomplete models. A Pearson correlation of 0.85 between QMEANDisCo global score and lDDT global score could be achieved in this case. A coverage corrected version of this plot is available as Figure 5 in the main manuscript.

Statistical Potential Parametrization

The probability density functions of the statistical potential terms are based on histograms with the following parametrization:

All-Atom Interaction Potential: minimal distance: 0.0Å, maximal distance: 10.0Å, bin size: 0.5Å, sequence separation: 4 residues
Cβ Interaction Potential: minimal distance: 0.0Å, maximal distance: 12.0Å, bin size: 0.5Å, sequence separation: 4 residues
Packing Potential: inclusion radius: 5.0Å, max counts: 32
Torsion Potential: bin size: 20°
Cβ Packing Potential: inclusion radius: 8.0Å, max counts: 20
Reduced Potential: Every residue is represented by its Cα position p and a directional component v = *norm(ca_pos - n_pos) + norm (ca_pos - c_pos)*. For interacting residues r_1 and r_2, we can define a line l between p_1 and p_2. The statistical potential then considers:

- dist: distance between p_1 and p_2
- alpha: angle between v_1 and l
- beta: angle between v_2 and l
- gamma: dihedral angle between $(p_1 + v_1, p_1, p_2, p_2 + v_2)$

minimal distance: 0.0Å, maximal distance: 10.0Å, distance bin size: 1Å, sequence separation: 4, angular bin size (alpha, beta): 22.5°, dihedral bin size (gamma): 45°

Running HHblits for Homology Detection

HHblits version 2.0.16 is used to detect homologues given a target sequence stored in target.fasta. A search profile (target.a3m) is generated with:

```
hhblits -e 0.001 -i target.fasta -oa3m target.a3m -d path_to_nrdb
```

The last argument specifies the database for sequence search. QMEANDisCo uses the nr20 database (12Aug11) provided by the Söding lab.

Homologues are identified using the following command:

```
hhblits -macct 0.35 -n 1 -e 0.001 -Z 10000 -B 10000 -i target.a3m -o
target.hhr -d hhblits_db
```

The hhblits_db is the profile database that is searched for homologues. QMEANDisCo uses the weekly updated SWISS-MODEL template library (SMTL) for homology search. The SMTL is a copy of the protein data bank (PDB) enriched with annotations relevant for modelling.

Clustering Homologues in DisCo Construction

Given *k* homologues with their respective pairwise sequence alignment towards a common target sequence (the sequence of the model being assessed), clustering proceeds as follows:

- Construct a multiple sequence alignment using the "star-method" with the target sequence as "center" sequence
- Calculate all $k * (k-1)/2$ pairwise sequence similarities. Given a substitution matrix S, the sequence similarity between sequences a and b in the multiple sequence alignment is defined as: $seqsim(a,b) = \frac{1}{N*(max(S)-min(S))} \sum_i S(a[i],\ b[i]) - min(S)$ where i iterates over all N pairs of characters in a and b that are aligned and covered by structural information (Cα position resolved). BLOSUM62 is used as substitution matrix S.
- Perform a hierarchical agglomerative clustering using an unweighted average linkage criterion for cluster merging.
- Stop merging clusters when distance between clusters falls below 0.5.

Feature Groups for Neural Network Training

Some quality predictors for the neural network input layer are potentially invalid. Examples include terminal residues for which no backbone dihedral angles can be estimated, invalid DisCo scores due to lacking HHblits search results or no agreement terms due to absent external dependencies. QMEANDisCo solves that problem with the NNScorer object that trains a neural network for each of the feature combinations specified below. Given a set of input features, the NNScorer iterates over the list of feature combinations and applies the first neural network for which all required input features are available. The used feature combinations are:

- avg_torsion, avg_reduced, avg_interaction, avg_cb_interaction, avg_packing, avg_cb_packing, N, packing, cb_packing, solv_acc, clash, torsion, reduced, interaction, cb_interaction, avg_ss_agreement, avg_acc_agreement, ss_agreement, acc_agreement, disco_related_features
- avg_torsion, avg_reduced, avg_interaction, avg_cb_interaction, avg_packing, avg_cb_packing, N, packing, cb_packing, solv_acc, clash, reduced, interaction, cb_interaction, avg_ss_agreement, avg_acc_agreement, ss_agreement, acc_agreement, disco_related_features
- avg_torsion, avg_reduced, avg_interaction, avg_cb_interaction, avg_packing, avg_cb_packing, N, packing, cb_packing, solv_acc, clash, torsion, avg_ss_agreement, avg_acc_agreement, ss_agreement, acc_agreement, disco_related_features
- avg_torsion, avg_reduced, avg_interaction, avg_cb_interaction, avg_packing, avg_cb_packing, N, packing, cb_packing, solv_acc, clash, torsion, reduced, interaction, cb_interaction, disco_related_features
- avg_torsion, avg_reduced, avg_interaction, avg_cb_interaction, avg_packing, avg_cb_packing, N, packing, cb_packing, solv_acc, clash, torsion, reduced, interaction, cb_interaction, avg_ss_agreement, avg_acc_agreement, ss_agreement, acc_agreement
- avg_torsion, avg_reduced, avg_interaction, avg_cb_interaction, avg_packing, avg_cb_packing, N, packing, cb_packing, solv_acc, clash, torsion, reduced, interaction, cb_interaction
- avg_torsion, avg_reduced, avg_interaction, avg_cb_interaction, avg_packing, avg_cb_packing, N, packing, cb_packing, solv_acc, clash, torsion, avg_ss_agreement, avg_acc_agreement, ss_agreement, acc_agreement
- avg_torsion, avg_reduced, avg_interaction, avg_cb_interaction, avg_packing, avg_cb_packing, N, packing, cb_packing, solv_acc, clash, torsion, disco_related_features
- avg_torsion, avg_reduced, avg_interaction, avg_cb_interaction, avg_packing, avg_cb_packing, N, packing, cb_packing, solv_acc, clash, reduced, interaction, cb_interaction, avg_ss_agreement, avg_acc_agreement, ss_agreement, acc_agreement
- avg_torsion, avg_reduced, avg_interaction, avg_cb_interaction, avg_packing, avg_cb_packing, N, packing, cb_packing, solv_acc, clash, reduced, interaction, cb_interaction, disco_related_features
- avg_torsion, avg_reduced, avg_interaction, avg_cb_interaction, avg_packing, avg_cb_packing, N, packing, cb_packing, solv_acc, clash, avg_ss_agreement, avg_acc_agreement, ss_agreement, acc_agreement, disco_related_features
- avg_torsion, avg_reduced, avg_interaction, avg_cb_interaction, avg_packing, avg_cb_packing, N, packing, cb_packing, solv_acc, clash, torsion
- avg_torsion, avg_reduced, avg_interaction, avg_cb_interaction, avg_packing, avg_cb_packing, N, packing, cb_packing, solv_acc, clash, reduced, interaction, cb_interaction
- avg_torsion, avg_reduced, avg_interaction, avg_cb_interaction, avg_packing, avg_cb_packing, N, packing, cb_packing, solv_acc, clash, avg_ss_agreement, avg_acc_agreement, ss_agreement, acc_agreement
- avg_torsion, avg_reduced, avg_interaction, avg_cb_interaction, avg_packing, avg_cb_packing, N, packing, cb_packing, solv_acc, clash, disco_related_features
- avg_torsion, avg_reduced, avg_interaction, avg_cb_interaction, avg_packing, avg_cb_packing, N, packing, cb_packing, solv_acc, clash

Neural Networks and Additional Features

Starting from the linear combination employed by QMEAN 3, the impact of using neural networks and gradually adding more features specific to QMEANDisCo are reported in Table S3. Training and validation in this analysis follow the cross-validation procedure described in section 2.5 of the main manuscript. The neural networks are trained in form of full NNScorer objects with the finally used training parameterization/ network topology. However, the number of input features gradually increases. This gives the following NNScorers with their respective feature groups:

basic (features from the current version of QMEAN):

- packing, solv_acc, torsion, interaction, cb_interaction, ss_agreement, acc_agreement
- packing, solv_acc, torsion, interaction, cb_interaction
- packing, solv_acc, torsion, ss_agreement, acc_agreement
- packing, solv_acc, interaction, cb_interaction, ss_agreement, acc_agreement
- packing, solv_acc, torsion
- packing, solv_acc, interaction, cb_interaction
- packing, solv_acc, ss_agreement, acc_agreement
- packing, solv_acc

plus_4 (same as before but added 4 QMEANDisCo specific features)

- N, packing, cb_packing, solv_acc, clash, torsion, reduced, interaction, cb_interaction, ss_agreement, acc_agreement
- N, packing, cb_packing, solv_acc, clash, torsion, reduced, interaction, cb_interaction
- N, packing, cb_packing, solv_acc, clash, torsion, ss_agreement, acc_agreement
- N, packing, cb_packing, solv_acc, clash, reduced, interaction, cb_interaction, ss_agreement, acc_agreement
- N, packing, cb_packing, solv_acc, clash, torsion
- N, packing, cb_packing, solv_acc, clash, reduced, interaction, cb_interaction
- N, packing, cb_packing, solv_acc, clash, ss_agreement, acc_agreement
- N, packing, cb_packing, solv_acc, clash

plus_avg (same as before but added average values of statistical potentials and agreement terms)

- avg_torsion, avg_reduced, avg_interaction, avg_cb_interaction, avg_packing, avg_cb_packing, N, packing, cb_packing, solv_acc, clash, torsion, reduced, interaction, cb_interaction, avg_ss_agreement, avg_acc_agreement, ss_agreement, acc_agreement
- avg_torsion, avg_reduced, avg_interaction, avg_cb_interaction, avg_packing, avg_cb_packing, N, packing, cb_packing, solv_acc, clash, torsion, reduced, interaction, cb_interaction
- avg_torsion, avg_reduced, avg_interaction, avg_cb_interaction, avg_packing, avg_cb_packing, N, packing, cb_packing, solv_acc, clash, torsion, avg_ss_agreement, avg_acc_agreement, ss_agreement, acc_agreement
- avg_torsion, avg_reduced, avg_interaction, avg_cb_interaction, avg_packing, avg_cb_packing, N, packing, cb_packing, solv_acc, clash, reduced, interaction, cb_interaction, avg_ss_agreement, avg_acc_agreement, ss_agreement, acc_agreement
- avg_torsion, avg_reduced, avg_interaction, avg_cb_interaction, avg_packing, avg_cb_packing, N, packing, cb_packing, solv_acc, clash, torsion
- avg_torsion, avg_reduced, avg_interaction, avg_cb_interaction, avg_packing, avg_cb_packing, N, packing, cb_packing, solv_acc, clash, reduced, interaction, cb_interaction
- avg_torsion, avg_reduced, avg_interaction, avg_cb_interaction, avg_packing, avg_cb_packing, N, packing, cb_packing, solv_acc, clash, avg_ss_agreement, avg_acc_agreement, ss_agreement, acc_agreement
- avg_torsion, avg_reduced, avg_interaction, avg_cb_interaction, avg_packing, avg_cb_packing, N, packing, cb_packing, solv_acc, clash,

full (The finally used full NNScorer with the previously defined feature groups)

Tab. S1. Top performing training parameters/network topologogies when validated on CAMEO and CASP12. This is the result of the first optimization round and thus excludes all data points where any of the input features is invalid. The network parameters are separated by '_' and encode 1. training data 2. optimizer 3. error function 4. training epochs 5. batch size 6. zero based topology index referring to the topologies listed in section 2.5 in the main manuscript.

	ROC AUC				Spearman Correlation				Pearson Correlation			
	CAMEO		CASP12		CAMEO		CASP12		CAMEO		CASP12	
Network Parameters	Overall	per-model	Overall	per-model	Overall	per-model	Overall	per-model	Overall	per-model	Overall	per-model
cameo_rmsprop_mse_200_200_0	0.935	0.876	0.941	0.831	0.862	0.648	0.861	0.576	0.881	0.679	0.864	0.592
mixed_rmsprop_mse_200_400_0	0.935	0.875	0.945	0.832	0.863	0.649	0.872	0.587	0.881	0.681	0.877	0.603
mixed_rmsprop_mse_100_100_0	0.936	0.875	0.943	0.831	0.865	0.649	0.869	0.584	0.882	0.681	0.875	0.601
mixed_rmsprop_mse_200_100_0	0.937	0.875	0.944	0.830	0.865	0.649	0.869	0.581	0.883	0.680	0.874	0.598
mixed_rmsprop_mse_100_400_2	0.936	0.875	0.943	0.828	0.864	0.648	0.870	0.581	0.882	0.680	0.874	0.597
cameo_rmsprop_mse_100_200_0	0.935	0.874	0.942	0.831	0.863	0.648	0.863	0.576	0.882	0.679	0.866	0.590
mixed_rmsprop_mse_100_400_0	0.936	0.874	0.945	0.831	0.865	0.646	0.871	0.587	0.882	0.678	0.877	0.603
mixed_adam_mse_100_100_0	0.935	0.874	0.944	0.829	0.862	0.646	0.871	0.582	0.880	0.678	0.876	0.598
mixed_rmsprop_mse_400_200_2	0.934	0.874	0.943	0.828	0.861	0.646	0.868	0.577	0.880	0.678	0.873	0.593
cameo_rmsprop_mse_100_400_2	0.934	0.874	0.940	0.830	0.860	0.645	0.860	0.576	0.880	0.678	0.864	0.589
mixed_rmsprop_mse_400_100_0	0.935	0.874	0.943	0.830	0.861	0.646	0.868	0.580	0.880	0.677	0.873	0.597
mixed_rmsprop_mse_100_100_2	0.936	0.874	0.944	0.828	0.864	0.647	0.871	0.580	0.882	0.679	0.875	0.596
cameo_rmsprop_mse_100_400_0	0.935	0.874	0.943	0.831	0.863	0.647	0.864	0.580	0.881	0.678	0.868	0.594
cameo_rmsprop_mse_400_200_0	0.935	0.874	0.941	0.829	0.860	0.647	0.860	0.573	0.880	0.677	0.864	0.588
mixed_rmsprop_mse_200_200_0	0.936	0.874	0.944	0.830	0.864	0.647	0.871	0.586	0.881	0.679	0.876	0.602
cameo_rmsprop_mse_200_400_2	0.934	0.874	0.941	0.829	0.858	0.646	0.860	0.574	0.878	0.677	0.865	0.589
mixed_rmsprop_mse_100_200_8	0.935	0.874	0.942	0.829	0.862	0.646	0.869	0.581	0.881	0.679	0.873	0.598
cameo_rmsprop_mse_200_400_0	0.935	0.874	0.941	0.830	0.860	0.647	0.862	0.578	0.880	0.678	0.866	0.592
cameo_adam_mse_200_200_0	0.935	0.874	0.942	0.831	0.860	0.644	0.861	0.576	0.879	0.675	0.864	0.590
cameo_rmsprop_mse_200_100_0	0.935	0.873	0.942	0.832	0.862	0.646	0.861	0.581	0.881	0.678	0.865	0.595
cameo_rmsprop_mse_100_200_2	0.935	0.873	0.941	0.829	0.861	0.644	0.861	0.575	0.881	0.676	0.865	0.591
cameo_adam_mse_400_100_0	0.934	0.873	0.942	0.832	0.859	0.647	0.861	0.579	0.879	0.677	0.866	0.594
mixed_rmsprop_mse_100_200_0	0.935	0.873	0.944	0.829	0.863	0.647	0.871	0.586	0.882	0.678	0.876	0.602
mixed_rmsprop_mse_100_200_2	0.936	0.873	0.943	0.828	0.863	0.645	0.869	0.577	0.881	0.677	0.873	0.594
mixed_adam_mse_200_400_0	0.935	0.873	0.941	0.827	0.860	0.644	0.867	0.583	0.879	0.676	0.871	0.599
mixed_adam_mse_400_400_0	0.935	0.873	0.941	0.830	0.862	0.643	0.865	0.582	0.880	0.675	0.870	0.599
mixed_rmsprop_mse_200_400_1	0.936	0.873	0.942	0.828	0.862	0.644	0.868	0.581	0.880	0.677	0.873	0.598
cameo_rmsprop_mse_400_100_0	0.934	0.873	0.941	0.833	0.860	0.644	0.860	0.575	0.879	0.675	0.865	0.590
cameo_adam_mse_100_400_0	0.935	0.873	0.942	0.832	0.861	0.644	0.860	0.576	0.880	0.676	0.864	0.590
mixed_rmsprop_mse_400_400_0	0.936	0.873	0.944	0.832	0.862	0.646	0.871	0.585	0.881	0.677	0.876	0.601
cameo_rmsprop_mse_100_100_0	0.935	0.873	0.941	0.829	0.861	0.646	0.861	0.577	0.880	0.678	0.865	0.592
cameo_adam_mse_100_200_0	0.934	0.873	0.941	0.829	0.860	0.644	0.862	0.581	0.879	0.676	0.864	0.594
cameo_rmsprop_mse_400_400_0	0.935	0.873	0.941	0.832	0.861	0.647	0.861	0.579	0.880	0.677	0.865	0.594
mixed_adam_mse_200_200_0	0.935	0.873	0.944	0.829	0.860	0.645	0.872	0.584	0.879	0.676	0.876	0.601
mixed_adam_mse_100_200_0	0.935	0.872	0.942	0.827	0.862	0.645	0.868	0.579	0.880	0.676	0.873	0.595
cameo_adam_mse_100_100_0	0.934	0.872	0.941	0.829	0.859	0.642	0.859	0.572	0.878	0.674	0.864	0.588
cameo_rmsprop_mse_100_100_2	0.933	0.872	0.940	0.829	0.858	0.643	0.859	0.575	0.878	0.675	0.864	0.590
cameo_adam_mse_400_400_0	0.934	0.872	0.940	0.830	0.857	0.641	0.858	0.575	0.877	0.673	0.863	0.590
mixed_rmsprop_absolute_100_100_0	0.934	0.872	0.943	0.827	0.864	0.652	0.871	0.587	0.881	0.681	0.874	0.601
mixed_rmsprop_mse_200_400_8	0.935	0.872	0.942	0.828	0.862	0.644	0.867	0.577	0.880	0.676	0.872	0.593
mixed_rmsprop_absolute_200_400_0	0.934	0.872	0.944	0.829	0.864	0.651	0.873	0.592	0.881	0.679	0.877	0.605
cameo_rmsprop_mse_400_100_2	0.934	0.872	0.941	0.828	0.857	0.643	0.860	0.571	0.878	0.673	0.864	0.587
mixed_adam_mse_100_400_0	0.934	0.872	0.943	0.829	0.859	0.644	0.870	0.581	0.879	0.676	0.874	0.598
mixed_rmsprop_mse_200_100_2	0.934	0.872	0.943	0.827	0.860	0.643	0.869	0.579	0.879	0.676	0.873	0.594
mixed_adam_mse_200_200_2	0.934	0.872	0.943	0.828	0.860	0.643	0.868	0.581	0.879	0.673	0.873	0.595
cameo_adam_mse_200_400_0	0.934	0.872	0.941	0.833	0.859	0.643	0.858	0.573	0.878	0.674	0.862	0.588
cameo_adam_mse_400_400_2	0.933	0.872	0.940	0.830	0.855	0.642	0.858	0.576	0.876	0.673	0.862	0.591
cameo_rmsprop_mse_100_400_1	0.934	0.871	0.941	0.832	0.859	0.642	0.859	0.577	0.879	0.675	0.863	0.591
mixed_rmsprop_mse_100_100_7	0.934	0.871	0.942	0.828	0.860	0.641	0.869	0.576	0.879	0.673	0.873	0.592
mixed_adam_mse_100_200_2	0.934	0.871	0.942	0.828	0.859	0.640	0.868	0.580	0.879	0.672	0.872	0.596
mixed_rmsprop_absolute_100_400_0	0.934	0.871	0.944	0.828	0.865	0.652	0.873	0.590	0.882	0.681	0.877	0.603
cameo_adam_mse_100_400_2	0.934	0.871	0.943	0.832	0.858	0.642	0.863	0.576	0.878	0.674	0.867	0.591
cameo_rmsprop_mse_400_400_2	0.933	0.871	0.941	0.829	0.856	0.640	0.860	0.571	0.877	0.673	0.865	0.586
cameo_rmsprop_absolute_100_400_0	0.934	0.871	0.943	0.830	0.864	0.651	0.865	0.581	0.882	0.679	0.869	0.595
cameo_adam_mse_400_200_0	0.933	0.871	0.942	0.829	0.856	0.642	0.862	0.573	0.877	0.675	0.867	0.588
mixed_rmsprop_mse_400_100_2	0.934	0.871	0.942	0.829	0.859	0.643	0.869	0.578	0.879	0.676	0.874	0.593
mixed_rmsprop_absolute_200_200_0	0.934	0.871	0.944	0.831	0.864	0.651	0.873	0.589	0.881	0.680	0.878	0.604
cameo_rmsprop_mse_200_200_2	0.934	0.871	0.941	0.832	0.859	0.641	0.860	0.576	0.878	0.673	0.864	0.590
cameo_rmsprop_mse_200_100_2	0.934	0.871	0.939	0.829	0.859	0.641	0.859	0.574	0.878	0.674	0.862	0.590

Tab. S2. Top performing training parameters/network topologogies when validated on CAMEO and CASP12. This is the result of the second optimization round where data points with invalid input features are handled with the NNScorer described in section 2.5 of the main manuscript. The finally used parametrization is highlighted with bold font. The network parameters are equally encoded as in Tab. S1.

	ROC AUC				Spearman Correlation				Pearson Correlation			
	CAMEO		CASP12		CAMEO		CASP12		CAMEO		CASP12	
Network Parameters	Overall	per-model	Overall	per-model	Overall	per-model	Overall	per-model	Overall	per-model	Overall	per-model
mixed_rmsprop_mse_200_200_0	0.940	0.874	0.945	0.815	0.873	0.654	0.833	0.559	0.889	0.686	0.855	0.569
mixed_rmsprop_mse_100_200_2	0.940	0.874	0.945	0.816	0.873	0.650	0.833	0.558	0.889	0.684	0.854	0.568
mixed_rmsprop_mse_100_200_0	0.940	0.874	0.947	0.816	0.873	0.651	0.834	0.559	0.889	0.683	0.857	0.571
**mixed_rmsprop_mse_100_400_0**	0.940	0.874	0.946	0.817	0.874	0.650	0.835	0.563	0.889	0.682	0.856	0.572
mixed_rmsprop_mse_100_400_2	0.940	0.874	0.945	0.815	0.874	0.650	0.833	0.556	0.889	0.683	0.855	0.567
cameo_rmsprop_mse_100_100_0	0.939	0.873	0.934	0.809	0.871	0.650	0.791	0.531	0.887	0.683	0.812	0.535
mixed_adam_mse_100_400_0	0.940	0.873	0.944	0.815	0.871	0.649	0.831	0.555	0.887	0.681	0.852	0.566
mixed_adam_mse_200_400_0	0.938	0.873	0.944	0.814	0.870	0.649	0.829	0.555	0.886	0.681	0.852	0.565
cameo_rmsprop_mse_100_200_0	0.940	0.873	0.934	0.806	0.872	0.649	0.792	0.530	0.887	0.680	0.811	0.532
mixed_rmsprop_mse_100_100_0	0.940	0.873	0.945	0.814	0.872	0.651	0.831	0.560	0.888	0.683	0.853	0.570
mixed_rmsprop_mse_200_400_0	0.940	0.873	0.945	0.816	0.873	0.648	0.832	0.559	0.889	0.680	0.853	0.569
cameo_rmsprop_mse_400_200_0	0.939	0.873	0.933	0.807	0.869	0.645	0.789	0.525	0.885	0.676	0.810	0.526
mixed_adam_mse_100_200_0	0.939	0.872	0.945	0.816	0.870	0.647	0.832	0.557	0.886	0.679	0.853	0.568
cameo_adam_mse_400_100_0	0.938	0.872	0.932	0.807	0.868	0.645	0.785	0.523	0.884	0.676	0.808	0.524
cameo_rmsprop_mse_200_400_0	0.939	0.872	0.932	0.805	0.870	0.647	0.789	0.526	0.886	0.679	0.810	0.528
mixed_rmsprop_mse_400_100_0	0.939	0.872	0.943	0.813	0.872	0.648	0.827	0.554	0.887	0.681	0.848	0.565
mixed_rmsprop_mse_100_200_8	0.939	0.872	0.944	0.816	0.871	0.649	0.832	0.557	0.887	0.681	0.853	0.567
cameo_rmsprop_mse_100_400_0	0.940	0.872	0.934	0.805	0.871	0.650	0.790	0.528	0.887	0.682	0.812	0.531
mixed_rmsprop_mse_200_400_8	0.940	0.872	0.945	0.815	0.873	0.646	0.832	0.561	0.888	0.680	0.854	0.570
cameo_rmsprop_mse_200_400_2	0.938	0.872	0.934	0.804	0.868	0.647	0.789	0.525	0.885	0.680	0.811	0.529
mixed_rmsprop_mse_100_100_2	0.940	0.872	0.945	0.814	0.872	0.647	0.834	0.558	0.888	0.681	0.856	0.567
cameo_rmsprop_mse_200_100_0	0.939	0.872	0.933	0.808	0.870	0.647	0.787	0.528	0.886	0.679	0.807	0.531
cameo_adam_mse_100_200_0	0.938	0.872	0.933	0.806	0.867	0.646	0.788	0.527	0.884	0.677	0.810	0.529
mixed_adam_mse_100_100_0	0.939	0.872	0.943	0.816	0.870	0.646	0.832	0.559	0.887	0.679	0.853	0.569
cameo_rmsprop_mse_100_400_1	0.939	0.872	0.933	0.807	0.871	0.645	0.792	0.533	0.886	0.677	0.812	0.535
mixed_rmsprop_mse_200_100_0	0.940	0.872	0.945	0.817	0.872	0.650	0.831	0.561	0.888	0.681	0.853	0.573
mixed_adam_mse_400_400_0	0.939	0.872	0.943	0.812	0.869	0.648	0.829	0.554	0.886	0.680	0.851	0.562
cameo_rmsprop_mse_400_400_0	0.938	0.871	0.932	0.807	0.869	0.647	0.785	0.528	0.885	0.677	0.807	0.530
mixed_rmsprop_mse_200_100_2	0.939	0.871	0.945	0.815	0.871	0.647	0.832	0.555	0.887	0.680	0.854	0.566
mixed_rmsprop_mse_200_400_1	0.938	0.871	0.943	0.811	0.869	0.646	0.830	0.553	0.886	0.680	0.851	0.563
mixed_rmsprop_mse_400_400_0	0.939	0.871	0.945	0.815	0.872	0.648	0.832	0.554	0.887	0.680	0.853	0.567
cameo_rmsprop_mse_100_200_2	0.939	0.871	0.934	0.806	0.870	0.644	0.795	0.533	0.886	0.677	0.816	0.537
mixed_rmsprop_mse_400_200_2	0.939	0.871	0.945	0.815	0.871	0.647	0.833	0.558	0.886	0.678	0.853	0.569
cameo_rmsprop_mse_200_200_0	0.939	0.871	0.933	0.806	0.870	0.648	0.789	0.526	0.886	0.679	0.809	0.528
mixed_rmsprop_absolute_100_400_0	0.938	0.871	0.946	0.818	0.873	0.656	0.840	0.572	0.889	0.685	0.860	0.580
cameo_adam_mse_100_100_0	0.939	0.871	0.933	0.804	0.869	0.644	0.790	0.527	0.884	0.675	0.809	0.529
cameo_adam_mse_200_200_0	0.938	0.871	0.931	0.804	0.867	0.644	0.784	0.528	0.884	0.675	0.804	0.529
cameo_adam_mse_200_400_0	0.938	0.871	0.931	0.805	0.868	0.643	0.780	0.527	0.884	0.675	0.803	0.527
cameo_rmsprop_mse_100_100_2	0.938	0.871	0.932	0.806	0.869	0.643	0.789	0.528	0.885	0.676	0.809	0.531
mixed_adam_mse_200_200_0	0.938	0.871	0.943	0.814	0.868	0.647	0.830	0.554	0.885	0.679	0.850	0.564
cameo_rmsprop_mse_200_200_2	0.938	0.871	0.932	0.807	0.868	0.645	0.788	0.529	0.884	0.676	0.807	0.531
cameo_adam_mse_400_400_0	0.938	0.871	0.933	0.808	0.867	0.643	0.785	0.526	0.883	0.674	0.806	0.528
mixed_adam_mse_200_200_2	0.939	0.870	0.944	0.814	0.869	0.646	0.830	0.556	0.886	0.678	0.851	0.565
mixed_rmsprop_mse_100_100_7	0.938	0.870	0.943	0.811	0.868	0.643	0.827	0.550	0.885	0.676	0.849	0.561
mixed_rmsprop_mse_400_100_2	0.938	0.870	0.944	0.813	0.868	0.644	0.830	0.555	0.885	0.677	0.852	0.567
cameo_adam_mse_100_400_2	0.937	0.870	0.930	0.803	0.864	0.642	0.788	0.529	0.882	0.673	0.808	0.531
cameo_rmsprop_mse_400_100_0	0.938	0.870	0.933	0.807	0.869	0.643	0.787	0.527	0.885	0.675	0.809	0.529
cameo_adam_mse_100_400_0	0.938	0.870	0.930	0.806	0.867	0.644	0.784	0.525	0.884	0.676	0.804	0.527
mixed_adam_mse_100_200_2	0.938	0.870	0.944	0.813	0.868	0.646	0.832	0.556	0.885	0.677	0.853	0.564
cameo_rmsprop_mse_100_400_2	0.938	0.870	0.933	0.805	0.867	0.645	0.789	0.526	0.884	0.677	0.811	0.529
mixed_rmsprop_absolute_200_200_0	0.939	0.869	0.944	0.814	0.873	0.653	0.835	0.566	0.889	0.681	0.854	0.574
cameo_rmsprop_mse_400_100_2	0.938	0.869	0.932	0.804	0.868	0.642	0.786	0.525	0.884	0.673	0.807	0.526
cameo_adam_mse_400_400_2	0.937	0.869	0.932	0.808	0.866	0.643	0.787	0.530	0.883	0.673	0.807	0.531
mixed_rmsprop_absolute_200_400_0	0.938	0.869	0.945	0.814	0.873	0.653	0.835	0.564	0.888	0.682	0.854	0.573
cameo_rmsprop_mse_200_100_2	0.938	0.868	0.933	0.805	0.867	0.639	0.788	0.532	0.883	0.671	0.808	0.534
mixed_rmsprop_absolute_100_100_0	0.939	0.868	0.946	0.814	0.874	0.653	0.837	0.563	0.889	0.681	0.857	0.572
cameo_rmsprop_mse_400_400_2	0.937	0.868	0.931	0.808	0.865	0.641	0.786	0.529	0.882	0.672	0.804	0.532
cameo_rmsprop_absolute_100_400_0	0.938	0.868	0.933	0.807	0.873	0.650	0.796	0.540	0.888	0.679	0.814	0.542
cameo_adam_mse_400_200_0	0.937	0.868	0.931	0.805	0.864	0.640	0.783	0.521	0.880	0.671	0.803	0.523

Tab. S3. Impact of using neural networks instead of linear combinations and gradually adding more features specific to QMEANDisCo. Predictors and evaluation procedure is described in supplemental section "Neural Networks and Additional Features".

	ROC AUC				Spearman Correlation				Pearson Correlation			
	CAMEO		CASP12		CAMEO		CASP12		CAMEO		CASP12	
Predictor	Overall	per-model	Overall	per-model	Overall	per-model	Overall	per-model	Overall	per-model	Overall	per-model
Linear Combination (QMEAN 3)	0.872	0.822	0.863	0.794	0.708	0.531	0.663	0.491	0.725	0.558	0.637	0.497
basic	0.878	0.824	0.878	0.784	0.722	0.534	0.704	0.490	0.756	0.563	0.711	0.500
plus_4	0.884	0.828	0.887	0.786	0.733	0.542	0.721	0.485	0.766	0.570	0.732	0.497
plus_avg	0.902	0.833	0.911	0.786	0.783	0.563	0.767	0.513	0.808	0.589	0.785	0.522
full (QMEANDisCo 3)	0.940	0.874	0.946	0.817	0.874	0.650	0.835	0.563	0.889	0.682	0.856	0.572

Tab. S4. Performances of the input features described in sections 2.3 and 2.4 of the main manuscript when applied on the data sets used for cross-validation

	ROC AUC				Spearman Correlation				Pearson Correlation			
	CAMEO		CASP12		CAMEO		CASP12		CAMEO		CASP12	
Feature	Overall	per-model	Overall	per-model	Overall	per-model	Overall	per-model	Overall	per-model	Overall	per-model
All-Atom Interaction Potential	0.799	0.733	0.779	0.725	-0.551	-0.327	-0.494	-0.289	-0.520	-0.342	-0.462	-0.303
CB Interaction Potential	0.734	0.638	0.736	0.640	-0.443	-0.186	-0.371	-0.153	-0.419	-0.199	-0.342	-0.162
Packing Potential	0.728	0.673	0.773	0.688	-0.403	-0.246	-0.516	-0.283	-0.455	-0.291	-0.448	-0.305
Torsion Potential	0.755	0.682	0.724	0.661	-0.481	-0.247	-0.345	-0.203	-0.475	-0.282	-0.332	-0.218
Solvent Accessibility Agreement	0.628	0.603	0.649	0.600	0.252	0.177	0.284	0.175	0.282	0.182	0.305	0.173
Secondary Structure Agreement	0.735	0.688	0.794	0.694	0.407	0.284	0.517	0.281	0.475	0.301	0.453	0.280
Solvent Accessibility	0.694	0.733	0.685	0.647	-0.381	-0.398	-0.370	-0.334	-0.423	-0.425	-0.385	-0.347
CB Packing Potential	0.704	0.631	0.742	0.643	-0.351	-0.187	-0.461	-0.233	-0.398	-0.234	-0.396	-0.255
Reduced Potential	0.752	0.668	0.778	0.677	-0.457	-0.232	-0.473	-0.214	-0.493	-0.266	-0.470	-0.233
Clash Score	0.567	0.520	0.538	0.521	-0.172	-0.043	-0.073	-0.004	-0.160	-0.074	-0.164	-0.048
N	0.654	0.706	0.650	0.625	0.304	0.359	0.319	0.317	0.316	0.368	0.297	0.313
DisCo	0.890	0.824	0.891	0.778	0.773	0.513	0.683	0.365	0.788	0.554	0.693	0.380

Tab. S5. Result of the full training parameter/network topology optimization described in section 2.5 of the main manuscript. The provided results are conceptually equal to the results in Tab. S2 with the difference that only CASP12 has been used for training.

	ROC AUC				Spearman Correlation				Pearson Correlation			
	CAMEO		CASP12		CAMEO		CASP12		CAMEO		CASP12	
Network Parameters	Overall	per-model	Overall	per-model	Overall	per-model	Overall	per-model	Overall	per-model	Overall	per-model
casp_rmsprop_mse_100_200_0	0.915	0.856	0.939	0.809	0.800	0.600	0.828	0.545	0.838	0.640	0.842	0.561
casp_rmsprop_absolute_100_200_0	0.923	0.855	0.943	0.808	0.821	0.607	0.836	0.552	0.855	0.644	0.854	0.568
casp_rmsprop_absolute_100_100_0	0.924	0.854	0.942	0.808	0.828	0.606	0.834	0.548	0.857	0.643	0.851	0.564
casp_rmsprop_absolute_100_400_2	0.922	0.853	0.940	0.800	0.821	0.604	0.833	0.542	0.852	0.643	0.845	0.555
casp_rmsprop_absolute_100_400_0	0.924	0.853	0.942	0.809	0.828	0.608	0.836	0.548	0.859	0.644	0.854	0.565
casp_rmsprop_mse_100_200_2	0.920	0.853	0.941	0.806	0.810	0.595	0.829	0.541	0.845	0.634	0.847	0.559
casp_rmsprop_mse_100_400_0	0.917	0.853	0.937	0.811	0.805	0.603	0.827	0.546	0.841	0.639	0.836	0.561
casp_rmsprop_absolute_100_400_1	0.923	0.852	0.943	0.805	0.827	0.603	0.834	0.547	0.858	0.642	0.851	0.561
casp_rmsprop_mse_200_400_0	0.915	0.852	0.938	0.801	0.802	0.597	0.826	0.534	0.838	0.632	0.842	0.549
casp_rmsprop_absolute_400_400_0	0.919	0.852	0.941	0.803	0.816	0.599	0.831	0.546	0.848	0.636	0.849	0.558
casp_rmsprop_mse_100_400_2	0.919	0.851	0.939	0.805	0.809	0.591	0.828	0.538	0.844	0.630	0.844	0.554
casp_rmsprop_mse_100_400_8	0.915	0.851	0.941	0.804	0.796	0.593	0.829	0.537	0.837	0.632	0.848	0.552
casp_adam_absolute_100_400_0	0.921	0.850	0.940	0.807	0.821	0.597	0.831	0.543	0.853	0.631	0.828	0.559
casp_rmsprop_absolute_100_200_1	0.921	0.850	0.938	0.797	0.822	0.595	0.829	0.539	0.852	0.633	0.845	0.554
casp_rmsprop_absolute_100_400_7	0.919	0.850	0.941	0.803	0.810	0.598	0.831	0.543	0.845	0.635	0.851	0.560
casp_rmsprop_absolute_200_200_0	0.919	0.850	0.940	0.803	0.817	0.597	0.830	0.542	0.849	0.635	0.847	0.559
casp_rmsprop_absolute_200_400_0	0.923	0.850	0.943	0.809	0.828	0.602	0.835	0.550	0.857	0.638	0.854	0.565
casp_rmsprop_mse_200_200_0	0.911	0.849	0.913	0.794	0.786	0.589	0.798	0.531	0.824	0.625	0.802	0.546
casp_rmsprop_mse_100_100_0	0.917	0.849	0.931	0.804	0.809	0.589	0.818	0.539	0.841	0.624	0.833	0.554
casp_rmsprop_absolute_100_200_2	0.916	0.849	0.941	0.804	0.798	0.599	0.833	0.544	0.839	0.638	0.851	0.559
casp_rmsprop_mse_200_100_0	0.914	0.849	0.936	0.801	0.805	0.589	0.822	0.535	0.833	0.624	0.836	0.550
casp_rmsprop_absolute_400_100_0	0.920	0.849	0.922	0.795	0.819	0.592	0.810	0.533	0.850	0.630	0.816	0.549
casp_rmsprop_absolute_100_400_8	0.920	0.848	0.938	0.805	0.818	0.595	0.829	0.545	0.851	0.635	0.844	0.559
casp_rmsprop_absolute_200_400_8	0.918	0.848	0.936	0.803	0.813	0.595	0.826	0.545	0.847	0.634	0.842	0.558
casp_adam_absolute_100_200_0	0.920	0.848	0.935	0.801	0.817	0.598	0.824	0.536	0.851	0.634	0.841	0.552
casp_rmsprop_mse_100_100_2	0.914	0.848	0.934	0.796	0.801	0.589	0.821	0.532	0.837	0.626	0.835	0.545
casp_rmsprop_absolute_100_200_7	0.917	0.847	0.942	0.802	0.801	0.589	0.832	0.537	0.839	0.627	0.842	0.554
casp_adam_absolute_200_400_0	0.917	0.847	0.938	0.799	0.814	0.594	0.824	0.533	0.846	0.626	0.841	0.550
casp_rmsprop_absolute_200_400_1	0.919	0.847	0.942	0.802	0.816	0.588	0.832	0.544	0.846	0.627	0.849	0.558
casp_rmsprop_absolute_100_200_8	0.921	0.847	0.943	0.801	0.823	0.593	0.833	0.538	0.854	0.633	0.852	0.557
casp_rmsprop_mse_100_400_1	0.917	0.847	0.939	0.803	0.808	0.587	0.828	0.533	0.842	0.625	0.844	0.551
casp_rmsprop_absolute_100_100_8	0.919	0.847	0.939	0.801	0.814	0.591	0.829	0.538	0.849	0.632	0.846	0.556
casp_rmsprop_absolute_200_400_2	0.915	0.846	0.938	0.804	0.798	0.589	0.827	0.542	0.836	0.628	0.841	0.556
casp_rmsprop_absolute_100_100_2	0.921	0.846	0.941	0.801	0.815	0.593	0.832	0.544	0.850	0.630	0.850	0.559
casp_adam_absolute_100_100_0	0.919	0.846	0.939	0.802	0.821	0.595	0.826	0.542	0.852	0.629	0.847	0.556
casp_adam_mse_100_100_0	0.917	0.846	0.932	0.793	0.809	0.582	0.818	0.521	0.842	0.618	0.832	0.538
casp_adam_mse_100_400_0	0.917	0.846	0.938	0.804	0.806	0.587	0.820	0.530	0.840	0.619	0.839	0.543
casp_rmsprop_mse_100_100_8	0.911	0.845	0.934	0.801	0.786	0.579	0.822	0.531	0.826	0.618	0.836	0.546
casp_rmsprop_absolute_200_200_2	0.914	0.845	0.939	0.802	0.795	0.586	0.827	0.541	0.834	0.624	0.846	0.555
casp_adam_mse_200_200_0	0.909	0.845	0.897	0.792	0.791	0.584	0.775	0.519	0.827	0.617	0.776	0.528
casp_rmsprop_mse_100_400_7	0.914	0.845	0.938	0.801	0.784	0.577	0.824	0.530	0.829	0.617	0.832	0.547
casp_rmsprop_absolute_200_100_0	0.912	0.845	0.936	0.802	0.792	0.590	0.826	0.541	0.828	0.624	0.836	0.555
casp_rmsprop_mse_200_200_2	0.914	0.844	0.933	0.794	0.797	0.581	0.821	0.532	0.836	0.621	0.834	0.546
casp_rmsprop_absolute_100_200_5	0.918	0.844	0.937	0.791	0.815	0.584	0.825	0.531	0.846	0.623	0.844	0.544
casp_rmsprop_absolute_100_400_5	0.914	0.844	0.938	0.794	0.804	0.583	0.827	0.533	0.832	0.624	0.846	0.548
casp_adam_absolute_200_100_0	0.918	0.844	0.937	0.797	0.815	0.588	0.827	0.541	0.845	0.622	0.842	0.552
casp_rmsprop_absolute_400_200_0	0.919	0.843	0.937	0.799	0.818	0.588	0.828	0.537	0.848	0.624	0.844	0.552
casp_rmsprop_mse_100_400_3	0.914	0.843	0.942	0.802	0.791	0.579	0.828	0.534	0.831	0.618	0.850	0.552
casp_rmsprop_absolute_200_400_7	0.916	0.843	0.940	0.802	0.801	0.584	0.830	0.537	0.840	0.621	0.848	0.553
casp_rmsprop_mse_100_100_1	0.912	0.843	0.936	0.800	0.792	0.575	0.824	0.531	0.831	0.613	0.841	0.546
casp_adam_absolute_100_200_1	0.910	0.843	0.931	0.799	0.796	0.587	0.818	0.536	0.830	0.620	0.832	0.547
casp_rmsprop_absolute_100_400_3	0.918	0.843	0.940	0.801	0.811	0.585	0.830	0.533	0.847	0.625	0.848	0.551
casp_rmsprop_mse_100_200_1	0.915	0.843	0.938	0.803	0.802	0.574	0.826	0.533	0.835	0.615	0.843	0.548
casp_rmsprop_absolute_400_200_8	0.915	0.843	0.937	0.796	0.809	0.581	0.828	0.539	0.841	0.619	0.838	0.549
casp_rmsprop_mse_200_400_2	0.912	0.843	0.929	0.793	0.796	0.577	0.813	0.526	0.830	0.616	0.831	0.539
casp_rmsprop_absolute_200_100_8	0.917	0.842	0.937	0.803	0.806	0.577	0.827	0.536	0.844	0.618	0.841	0.548
casp_rmsprop_mse_100_200_8	0.910	0.841	0.935	0.797	0.789	0.573	0.826	0.534	0.828	0.615	0.842	0.547
casp_rmsprop_absolute_100_100_7	0.916	0.841	0.940	0.798	0.802	0.579	0.830	0.530	0.840	0.616	0.848	0.549
casp_rmsprop_absolute_100_200_3	0.919	0.841	0.941	0.804	0.808	0.582	0.828	0.535	0.844	0.620	0.849	0.551
casp_rmsprop_mse_200_400_1	0.917	0.841	0.930	0.796	0.807	0.573	0.817	0.525	0.840	0.610	0.830	0.538
casp_adam_mse_400_100_0	0.914	0.840	0.933	0.798	0.805	0.575	0.815	0.522	0.834	0.605	0.834	0.536
casp_rmsprop_absolute_200_200_7	0.916	0.840	0.935	0.795	0.804	0.576	0.824	0.529	0.840	0.613	0.840	0.543

casp_rmsprop_absolute_100_100_1	0.918	0.840	0.942	0.801	0.810	0.578	0.833	0.536	0.847	0.618	0.851	0.553
casp_adam_absolute_400_200_0	0.910	0.840	0.935	0.787	0.789	0.579	0.823	0.521	0.827	0.611	0.840	0.535
casp_adam_mse_100_200_0	0.906	0.840	0.889	0.787	0.779	0.574	0.768	0.516	0.803	0.609	0.768	0.529
casp_adam_absolute_200_100_8	0.912	0.839	0.930	0.793	0.801	0.578	0.819	0.523	0.837	0.613	0.830	0.539
casp_rmsprop_mse_100_200_3	0.911	0.839	0.933	0.796	0.780	0.566	0.817	0.523	0.821	0.605	0.836	0.538
casp_rmsprop_absolute_400_400_2	0.911	0.839	0.932	0.794	0.793	0.578	0.823	0.531	0.830	0.612	0.825	0.544
casp_rmsprop_absolute_400_400_1	0.914	0.838	0.941	0.804	0.793	0.573	0.831	0.537	0.837	0.611	0.849	0.553
casp_rmsprop_absolute_200_100_1	0.911	0.838	0.939	0.802	0.792	0.572	0.829	0.536	0.829	0.606	0.845	0.552
casp_adam_mse_100_200_2	0.913	0.837	0.928	0.793	0.793	0.574	0.814	0.525	0.833	0.607	0.828	0.537
casp_adam_mse_400_400_0	0.911	0.837	0.932	0.790	0.795	0.567	0.810	0.514	0.828	0.604	0.823	0.530
casp_rmsprop_absolute_200_200_1	0.917	0.836	0.937	0.796	0.805	0.573	0.827	0.536	0.844	0.611	0.842	0.550
casp_rmsprop_mse_200_200_7	0.908	0.836	0.939	0.799	0.777	0.563	0.822	0.524	0.814	0.602	0.836	0.540
casp_rmsprop_absolute_200_100_2	0.910	0.835	0.930	0.796	0.789	0.576	0.819	0.535	0.828	0.611	0.833	0.545
casp_rmsprop_absolute_200_200_8	0.911	0.835	0.939	0.798	0.796	0.567	0.829	0.534	0.834	0.609	0.847	0.551

Tab. S6. Performance evaluation on CAMEO data (data collected during 12 weeks, CAMEO QE-submission dates from 2018-12-01 to 2019-03-02)

Method	ROC AUC		abs(Spearman Correlation)		abs(Pearson Correlation)	
	Overall	per-model	Overall	per-model	Overall	per-model
EQuant 2	0.704	0.715	0.366	0.319	0.434	0.376
Baseline Potential	0.771	0.712	0.474	0.278	0.498	0.314
VoroMQA_sw5	0.808	0.774	0.524	0.397	0.596	0.447
VoroMQA_v2	0.817	0.766	0.550	0.392	0.578	0.416
ProQ2	0.855	0.786	0.611	0.439	0.633	0.467
ProQ3D	0.856	0.804	0.608	0.461	0.419	0.500
QMEAN 3	0.870	0.826	0.669	0.521	0.721	0.562
ModFOLD6	0.878	0.837	0.660	0.527	0.668	0.583
ProQ3	0.882	0.821	0.659	0.489	0.442	0.485
ProQ3D_LDDT	0.897	0.837	0.705	0.548	0.763	0.598
ModFOLD7_lDDT	0.903	0.844	0.711	0.529	0.782	0.615
QMEANDisCo 2.5.1	0.934	0.868	0.826	0.594	0.857	0.645
QMEANDisCo 3	0.942	0.876	0.845	0.644	0.883	0.685

Tab. S7. Performance evaluation on the full CASP13 data set (submission stage 2) and on a high quality subset of the same data only originating from models with global lDDT > 0.6. Results for QMEANDisCo 3 have been generated retrospectively. When constructing DisCo, we excluded all homologues released after the CASP13 submission deadline of the corresponding EMA targets.

	ROC AUC				abs(Spearman Correlation)				abs(Pearson Correlation)			
	Full Data Set		High Quality Subset		Full Data Set		High Quality Subset		Full Data Set		High Quality Subset	
Method	Overall	per-model	Overall	per-model	Overall	per-model	Overall	per-model	Overall	per-model	Overall	per-model
3DCNN	0.753	0.709	0.741	0.750	0.489	0.366	0.395	0.367	0.109	0.331	0.406	0.400
MULTICOM-NOVEL	0.781	0.618	0.652	0.644	0.550	0.211	0.205	0.200	0.516	0.216	0.257	0.248
RaptorX-DeepQA	0.794	0.708	0.742	0.784	0.592	0.414	0.319	0.399	0.271	0.395	0.274	0.465
MASS1	0.799	0.682	0.769	0.759	0.595	0.370	0.417	0.363	0.436	0.367	0.428	0.423
MASS2	0.799	0.682	0.769	0.759	0.595	0.370	0.417	0.364	0.438	0.367	0.428	0.423
NAIVE	0.815	NaN	0.598	NaN	0.608	NaN	0.129	NaN	0.594	NaN	0.094	NaN
VoroMQA-B	0.821	0.688	0.776	0.762	0.633	0.385	0.420	0.357	0.474	0.363	0.433	0.415
VoroMQA-A	0.822	0.691	0.778	0.764	0.631	0.389	0.416	0.355	0.466	0.365	0.432	0.413
Pcons	0.830	0.704	0.731	0.801	0.651	0.416	0.307	0.466	0.625	0.425	0.247	0.553
ProQ3D-TM	0.831	0.715	0.778	0.797	0.652	0.418	0.443	0.422	0.386	0.423	0.369	0.509
SASHAN	0.833	0.694	0.753	0.735	0.656	0.373	0.415	0.361	0.559	0.364	0.435	0.390
ProQ4	0.845	0.740	0.789	0.797	0.689	0.456	0.446	0.437	0.640	0.467	0.475	0.495
ProQ2	0.848	0.697	0.769	0.777	0.673	0.378	0.403	0.394	0.548	0.378	0.414	0.458
Davis-EMAconsensus	0.852	0.747	0.766	0.823	0.698	0.487	0.363	0.477	0.532	0.480	0.345	0.566
ModFOLDclust2	0.857	0.747	0.783	0.824	0.700	0.490	0.405	0.486	0.657	0.499	0.336	0.577
Wallner	0.859	0.726	0.739	0.831	0.712	0.447	0.314	0.485	0.677	0.464	0.376	0.586
ProQ3D	0.862	0.725	0.799	0.800	0.701	0.431	0.471	0.439	0.348	0.419	0.332	0.522
UOSHAN	0.863	0.730	0.803	0.807	0.709	0.465	0.486	0.478	0.662	0.469	0.472	0.553
ModFOLD7	0.867	0.741	0.790	0.824	0.717	0.478	0.437	0.473	0.679	0.492	0.398	0.565
ModFOLD7_rank	0.867	0.741	0.790	0.824	0.717	0.478	0.437	0.473	0.679	0.492	0.398	0.565
Pcomb	0.868	0.750	0.757	0.840	0.740	0.506	0.345	0.517	0.666	0.522	0.425	0.612
LamoureuxLab	0.875	0.716	0.749	0.782	0.708	0.403	0.415	0.404	0.709	0.427	0.439	0.477
ModFOLD7_cor	0.876	0.768	0.788	0.841	0.742	0.510	0.447	0.518	0.679	0.537	0.384	0.616
ProQ3	0.878	0.735	0.817	0.816	0.714	0.450	0.496	0.466	0.357	0.380	0.286	0.504
ProQ3D-CAD	0.894	0.769	0.842	0.830	0.738	0.483	0.582	0.528	0.741	0.495	0.620	0.578
ProQ3D-lDDT	0.901	0.772	0.838	0.847	0.766	0.520	0.591	0.558	0.768	0.537	0.621	0.623
CPClab	0.909	0.793	0.847	0.854	0.718	0.446	0.606	0.578	0.741	0.472	0.636	0.638
FaeNNz	0.933	0.783	0.876	0.842	0.824	0.537	0.730	0.600	0.833	0.546	0.742	0.637
QMEANDisCo3	0.934	0.792	0.880	0.862	0.824	0.538	0.719	0.607	0.833	0.549	0.737	0.649

Tab. S8. Model length dependent estimation of expected prediction error based on global CAMEO evaluation. For each model length l, data points from models of length l +- 40 are considered.

Model Length	N Data Points	Expected Error	Model Length	N Data Points	Expected Error
40	881	0.115	225	1198	0.056
45	970	0.115	230	1207	0.055
50	1040	0.112	235	1208	0.054
55	1075	0.111	240	1196	0.051
60	1152	0.109	245	1185	0.052
65	1238	0.108	250	1179	0.053
70	1359	0.107	255	1187	0.052
75	1346	0.105	260	1193	0.052
80	1396	0.096	265	1195	0.051
85	1505	0.093	270	1212	0.051
90	1531	0.092	275	1201	0.05
95	1535	0.088	280	1221	0.051
100	1516	0.08	285	1256	0.051
105	1585	0.078	290	1247	0.052
110	1575	0.078	295	1193	0.052
115	1560	0.078	300	1183	0.052
120	1524	0.076	305	1222	0.052
125	1544	0.073	310	1215	0.052
130	1601	0.072	315	1204	0.053
135	1618	0.071	320	1146	0.053
140	1587	0.071	325	1135	0.052
145	1518	0.067	330	1114	0.051
150	1460	0.067	335	1066	0.052
155	1430	0.066	340	1045	0.051
160	1409	0.066	345	999	0.051
165	1339	0.067	350	933	0.051
170	1330	0.066	355	908	0.05
175	1315	0.066	360	864	0.049
180	1256	0.063	365	827	0.05
185	1207	0.063	370	805	0.049
190	1241	0.061	375	815	0.05
195	1249	0.06	380	782	0.05
200	1249	0.059	385	703	0.05
205	1203	0.059	390	671	0.052
210	1159	0.059	395	655	0.053
215	1157	0.058	400	667	0.053
220	1193	0.057			
